# Women’s leadership development programmes and gender equality: a systematic review in healthcare, academia and beyond

**DOI:** 10.1016/j.eclinm.2026.104185

**Published:** 2026-09-01

**Authors:** Daisy Morris, Sana Rauf, Kathleen Riach, Helena Teede

**Affiliations:** a Faculty of Medicine, Nursing and Health Sciences, Monash University, Melbourne, Victoria, Australia; b Adam Smith Business School, University of Glasgow, Glasgow, Scotland, United Kingdom

**Keywords:** Women’s leadership development programmes, Gender equity, Leadership outcomes, Systematic review, Kirkpatrick’s framework

## Abstract

**Background:**

Women comprise 70% of the global health workforce yet hold only around 25% of leadership positions. Women's leadership development programmes (WLDPs) are widely implemented as organisational equity strategies; this review examined whether their reported outcomes extend beyond individual development to measurable organisational change.

**Methods:**

Systematic review without meta-analysis (PROSPERO: CRD42024554419), reported in accordance with PRISMA and SWiM. Nine databases were searched from 25 September 2015 to 5 July 2026. We included peer-reviewed studies evaluating structured WLDPs delivered to adult professionals in formal organisational roles that reported outcomes for women. Student-only populations and mentorship- or coaching-only interventions were excluded. Summary data were extracted from published reports by two independent reviewers using a standardised template incorporating TIDieR intervention-description items; quality was assessed using design-appropriate Joanna Briggs Institute critical appraisal tools. Outcomes were mapped using an extended Kirkpatrick framework spanning individual to organisational outcomes.

**Findings:**

From 2314 records, 57 studies evaluating 52 WLDPs across 23 countries were included. Most studies were from the United States (n = 36). Designs were predominantly mixed-methods or qualitative; none used randomisation, and four included comparison groups. All studies reported individual-level outcomes, most commonly self-reported improvements in confidence, knowledge, leadership capability, and behaviour. Thirty-two studies reported any organisational outcome, usually promotion or retention indicators. Subjective measures predominated over objective outcomes. Demographic reporting was limited: only 22 studies reported race or ethnicity, and very few reported sexual orientation, disability, or disaggregated advancement outcomes.

**Interpretation:**

WLDPs appear to support individual development, but current evidence cannot establish organisational transformation. Their broader equity potential depends on structural accountability: transparent and equitable recruitment pathways, institutional investment, and disaggregated outcome reporting, a reorientation of responsibility from individuals to organisations.

**Funding:**

The Australian National Health and Medical Research Council (NHMRC).

**Research in context**

**Evidence before this study**

We searched nine bibliographic databases including Applied Social Sciences Index and Abstracts, Business Source Complete, CINAHL, Embase, ERIC, Ovid MEDLINE, PsycINFO, Scopus, and Web of Science from 25 September 2015 to 5 July 2026 using terms for women, leadership development programmes, and evaluation or outcomes. We included English-language, peer-reviewed evaluations of structured WLDPs delivered to adult professionals across sectors, and excluded student-only populations and mentorship- or coaching-only interventions. Previous reviews were limited by heterogeneous programme definitions, predominantly qualitative or descriptive evidence, and limited assessment of organisational outcomes; meta-analysis was not undertaken. Qualitative reviews have highlighted participant-perceived value but could not address causal attribution or equity effects. No prior synthesis has systematically mapped outcomes across both individual and organisational levels using an established evaluation framework, examined the equity of access to these programmes, or assessed whether evaluation designs are matched to the organisational change goals WLDPs are positioned to achieve.

**Added value of this study**

This review provides the first comprehensive, cross-sector synthesis of WLDP evaluations mapped against an extended Kirkpatrick framework distinguishing subjective from objective outcomes across seven levels, with sensitivity and exploratory analyses examining the presence of a pre-programme baseline, a non-participant comparison group, risk of bias, long-term follow-up, reported organisational embedding or support, and restriction to pre-post and controlled designs. All 57 included studies reported individual-level outcomes; 32 studies (56%) captured any organisational measure, typically via descriptive, non-attributed metrics. Where recruitment was described, selective entry predominated (37/43 studies), and demographic reporting was sparse: race or ethnicity was reported in 22 of 57 studies and outcomes were disaggregated by race or ethnicity in only one. We interpret these patterns as an equity paradox in reported access and evaluation: programmes framed as gender equity interventions frequently used selective entry processes, while demographic and disaggregated outcome reporting remained sparse. We also identified an implementation-evaluation gap, whereby programmes positioned as broader equity strategies were predominantly delivered to individuals and evaluated through individual-level outcomes.

**Implications of all the available evidence**

The majority of WLDPs as currently designed and evaluated do not appear to function as organisational equity strategies, indicating the need for reconceptualisation. Achieving Sustainable Development Goal 5 requires aligning WLDP design, implementation, and evaluation at organisational level, alongside broader organisational equity strategies. Accountability mechanisms for WLDPs could include transparent, equitable and accessible recruitment, together with outcome reporting disaggregated by race, career stage, and other intersectional dimensions. Institutions commissioning WLDPs must accept shared responsibility for the structural conditions that enable or constrain translating individual gains into systemic change.

## Introduction

The quality and equity of healthcare is largely influenced by who leads it. Gender-diverse leadership teams are associated with improved patient outcomes, stronger healthcare quality, and enhanced organisational effectiveness in healthcare.^1^, ^2^, ^3^ Yet despite women comprising approximately 70% of the global health workforce and 41% of active researchers, they only hold around 25% of leadership and senior roles.^4^, ^5^, ^6^, ^7^ This disparity persists against a backdrop of international commitments to gender equality, defined as equal rights and opportunities, under Sustainable Development Goal 5 (SDG5),^8^ and is sustained by multiple reinforcing barriers spanning individual, organisational, and systemic levels.^8^, ^9^, ^10^, ^11^ In an era of complex global challenges, diverse leadership is increasingly recognised as strategically necessary for navigating uncertainty, driving innovation, and delivering care that reflects the populations served.^12^^,^^13^

Addressing this requires moving beyond individual skill-building towards organisational and systemic strategies that create genuine motivation and opportunity for women's advancement at scale.^10^^,^^14^, ^15^, ^16^ Gender equity, defined as the processes and practices required to create gender equality,^17^ demands that women's leadership development be understood within its organisational context, where formal opportunity structures intersect with informal power structures.^18^, ^19^, ^20^ Women’s leadership development programmes (WLDPs) build individual leadership capability, and are increasingly positioned as interventions capable of driving organisational change.^21^^,^^22^ Realising this means challenging discriminatory practices, institutional culture, and accountability mechanisms that shape who leads health systems, and ultimately, who they serve.^22^, ^23^, ^24^, ^25^

Despite widespread WLDP implementation across healthcare, academic, and other sectors, evidence of organisational transformation remains fragmented. Evaluations frequently focus on individual participant satisfaction and self-reported outcomes, with less consistent measurement of behavioural transfer and organisational change.^22^^,^^26^^,^^27^ Studies predominantly examine isolated interventions within specific contexts,^9^^,^^28^^,^^29^ use heterogeneous outcomes, and predominantly qualitative evaluations,^27^ limiting comparability, generalisability, and producing mixed findings regarding broader institutional impact. Consequently, existing reviews have offered limited insight into whether observed benefits translate into organisational or equity gains, leaving organisations without clear evidence to guide investment.

Given the proliferation of WLDPs^30^ and their potential contribution to SDG5 and healthcare quality, robust evidence synthesis is needed. Because WLDP evidence is not confined to healthcare, this systematic review aims to synthesise WLDP evaluations across all sectors or disciplinary fields (noting evidence concentrates in academic and health) and assess whether reported outcomes extend beyond individual development to measurable organisational change. A multisector synthesis is useful not because leadership development is sector-neutral, but because it enables healthcare systems to learn from the broader organisational evidence while identifying what is required for WLDPs to contribute to measurable gender-equity change.

## Methods

This is a systematic review without meta-analysis, providing a narrative synthesis^31^ of heterogeneous WLDP evaluations for which a summary effect estimate was not appropriate. Narrative synthesis is suited to integrating complex evidence and generating analytically robust, policy-relevant insights where outcomes are context-dependent rather than driven by linear cause-effect relationships.^27^^,^^32^, ^33^, ^34^ The review protocol was registered a priori on PROSPERO (https://www.crd.york.ac.uk/PROSPERO/view/CRD42024554419) and conducted in accordance with PRISMA guidelines and the Synthesis Without Meta-analysis (SWiM) reporting guideline (Supplementary Table S1). The registered record was subsequently updated to operationalise the broad registered aim. Eligibility criteria were specified as set out below; narrative synthesis without meta-analysis was adopted in response to heterogeneity; JBI tools replaced CASP for quality appraisal; and outcomes were mapped using an extended Kirkpatrick framework. During peer review the search was expanded from five to nine databases and extended to July 2026, with records screened in duplicate (DM, SR) following the original procedures, yielding four additional included studies.^35^, ^36^, ^37^, ^38^

### Search strategy and selection criteria

Eligibility was based on the Population, Intervention, Comparator, Outcome (PICO) framework. We included peer-reviewed studies evaluating structured WLDPs delivered to adult professionals employed in formal organisational roles in all sectors/disciplinary fields or settings, published in English between 25 September 2015 and 5 July 2026. This start date aligns with UN SDG5 adoption, and captures programmes implemented under explicit international gender-equality commitments; earlier evidence has been synthesised elsewhere.^9^^,^^27^^,^^32^ Studies were required to report at least one evaluative outcome for women, including measurable advancement indicators (e.g., promotion, appointments), or theory-linked proximal indicators (e.g., leader identity, self-efficacy). All study designs reporting evaluative data were eligible. Studies were excluded if they enrolled student-only populations, for whom advancement outcomes such as promotion are not observable and organisational attribution is not possible, or if the intervention comprised mentorship or coaching only, since outcomes cannot be reliably attributed to structured programme components. Grey literature, trial registries, and unpublished studies were not systematically searched. No study authors were contacted for additional data. Sector or disciplinary field was defined as the broad area in which the programme operated (e.g., healthcare, public administration), and setting as the type of organisation delivering or hosting the programme (e.g., university, hospital, professional association).

Nine databases were searched from 25 September 2015 to 5 July 2026: Applied Social Sciences Index and Abstracts, Business Source Complete, CINAHL, Embase, ERIC, Ovid MEDLINE, PsycINFO, Scopus, and Web of Science. The search strategy was developed iteratively using controlled vocabulary and proximity operators combining terms for women, leadership development programmes, and outcomes or evaluation; full search terms for Ovid MEDLINE are provided in Supplementary Table S2. Additional studies were identified through forward and backward citation chaining of included articles.

Two reviewers (DM, SR) independently screened titles and abstracts and then full texts, with discrepancies resolved by discussion with a third reviewer (HT) using the Covidence web-based software. Inter-rater agreement was calculated using Cohen’s κ from the two reviewers’ independent decisions before consensus resolution at the title and abstract screening, full-text screening, and quality-appraisal stages. Data were extracted independently by two reviewers using a standardised template covering study characteristics, programme design and delivery, and reported outcomes. AI-assisted tools were not used in any part of the search, screening, or data extraction process.

### Ethics

Ethical approval was not required because this systematic review used data from published literature.

### Data and analysis

Two reviewers independently extracted data on study and programme characteristics structured according to the Template for Intervention Description and Replication (TIDieR) informed categories (Table 1 and Supplementary Table S3, with TIDieR mapping in Supplementary Table S4); recruitment and access models guided by the PROGRESS-Plus framework for equity-relevant characteristics (Supplementary Table S5); and outcome data aligned with the primary research question: whether WLDPs produce evidence of organisational change beyond individual development (Supplementary Table S6).

**Table 1:** Characteristics of included studies and programmes.

Author, year, country/ region	Study design	Sector/field	Programme name (Duration)	N and participant description	Data collection timepoints
Adeola, 2024, USA	Descriptive mixed-methods programme evaluation	Academic nursing	Midwest Nursing Research Society Leadership Academy (11 months)	N = 5 nurse scholars	Post-imm
Barnard, 2022, United Kingdom	Mixed-methods longitudinal study	Academic higher education	Aurora: Advance HE Women’s Leadership Program (several months)	N = 1094 early- or mid-career women working in UK higher education	Pre; Post-short (3–6 months; 15–18 months); Post-long (≥3 years)
Brixey, 2025, USA	Quantitative pre–post survey	Academic medicine	Launching a Women-Focused Faculty and Trainee Development Program (LAWFFT-UP) (21 months)	N = 35 academic health faculty and trainees	Pre; Post-imm
Brue, 2016, USA	Qualitative phenomenological study	Academic STEM	Oklahoma Career Tech Women in Leadership Program (8 months)	N = 7 women programme alumnae with substantial professional experience	Retrospective post-only; timing not specified
Brue, 2018, USA	Qualitative phenomenological follow-up study	Academic STEM	Oklahoma Career Tech Women in Leadership Program (8 months)	N = 7 women programme alumnae	Retrospective post-only; timing not specified
Chang, 2016, USA	Quantitative cohort comparison study	Academic medicine	AAMC Early- and Mid-Career Women in Medicine; Hedwig van Ameringen Executive Leadership in Academic Medicine (4 days for EWIM/MWIM; 1 year for ELAM)	N = 3268 women faculty	Post-long (annual tracking for up to 10 years)
Chang, 2020, USA	Quantitative retrospective cohort study	Academic medicine	Early- and Mid-Career Programs and ELAM (4 days for EWIM/MWIM; 1 year for ELAM)	N = 2719 women faculty at Assistant or Associate Professor level	Pre; Post-long (5 years; 10 years)
Chasserio, 2024, France	Mixed qualitative design	Professional, cross-sector	JUMP (8 months)	N = 47 women participants	Pre; Post-short (6 months)
Chaudron, 2021, USA	Quantitative pre–post self-assessment	Academic medicine	Developing from Within (DFW) Career Development Program (4 days)	N = 51 mid-career women across four cohorts, primarily medical faculty	Pre; Post-imm; Post-long (1 year)
Cordeiro, 2024, USA and LMICs	Mixed-methods explanatory sequential study	Academic medicine and global health	Female Global Scholars Program (2 years)	N = 22 women programme participants	Retrospective post-only
Danhauer, 2019, USA	Descriptive mixed-methods evaluation	Academic medicine	Early Career Development Program for Women Faculty (6 months)	N = 81 participants; n = 65 included in pre–post analysis; early-career women faculty, primarily at Assistant Professor level	Pre; Post-imm
David, 2025, USA	Retrospective cross-sectional survey evaluation	Academic medicine	Women’s Leadership Development Program at Yale School of Medicine (one semester)	N = 26 early-career women faculty across eight cohorts	Retrospective post-only
DeFrank-Cole, 2017, USA	Mixed-methods evaluation	Academic higher education	Women’s Leadership Initiative (15 weeks, with additional activities across the year)	N = 116 women faculty and staff across three cohorts	Pre; Post-imm
Deutsch, 2022, USA	Mixed-methods repeated cross-sectional study	Academic medicine	Women’s Empowerment and Leadership Initiative (WELI) (ongoing; evaluated over 14 months)	Baseline: n = 92/152; 6 months: n = 82/161; 14 months: n = 96/185; WELI members including advisors, protégés and dual protégé-advisors	Mid/post-enrolment; repeated follow-up (6 mo; 14 mo)
Drake, 2023, USA	Mixed descriptive case study	Academic medicine	Academic Career Leadership Academy in Medicine (ACCLAIM) (9 months)	N = 111 academic medical centre faculty scholars across nine cohorts	Pre; Post-imm
Fassiotto, 2024, USA	Longitudinal single-cohort programme evaluation	Academic medicine	Stanford Network for Advancement and Promotion (SNAP) (ongoing; evaluated over 5 years)	N = 10 women physicians from eight departments at Stanford Medicine	Post-long (annual surveys, Years 1–5)
Ford, 2021, USA	Quantitative longitudinal follow-up study	Academic medicine and STEM	ELAM and ELATES (12 months)	N = 287 academic women from medicine and STEMM disciplines	Pre; Post-short (6–8 weeks); Post-long (2 years)
Grando, 2022, USA	Mixed-methods programme evaluation	Academic medicine and biomedical informatics	Women in American Medical Informatics Association Leadership Program (1 year)	N = 24 women informatics professionals working primarily in academic roles	Pre; Post-session; Post-imm
Heilemann, 2024, Australia	Qualitative longitudinal interview study	Public sector: policing	Not reported (four sessions; total duration not reported)	N = 31 women Senior Sergeants and Inspectors	Post-short (1 mo; 4 mo; 9 mo)
Helitzer, 2016, USA and Canada	Qualitative narrative interview study	Academic medicine	EWIM, MWIM and ELAM Programs (3-day intensive programmes plus seminars; total duration not reported)	N = 45 women medical faculty	Post-long (retrospective alumnae assessment; intervals extending ≥1 year)
Herbst, 2024, South Africa	Mixed-methods evaluation	Academic higher education	Not reported (1 year)	N = 70 women academics across two cohorts	Pre; Mid; Post-imm
Hopkins, 2022, USA	Mixed-methods programme assessment	Nonprofit and human services	Leadership and Management Certificate Program (self-paced; total duration not fixed)	N = 76 women professionals, primarily in management roles	Retrospective/post-only
Jones, 2025, USA	Qualitative interviews and focus groups	Academic medicine	Engaging Peer Mentors for Opportunity, Well-Being, and Equity Realization (EMPOWER) (approximately 12 months)	N = 48 informants: women faculty leaders and programme advisors	Mid; Post-imm
Jowett, 2024, United Kingdom	Qualitative interview study	Sport: coaching	Female Coaches and High-Performance Leadership Programme (6 months)	N = 20 programme cohort; n = 17 evaluation participants; professional coaches with varied coaching experience	Post-imm; Post-long (3 years)
Kang, 2024, USA	Quantitative pre–post evaluation	Academic medicine	Reflect, Inspire, Strengthen and Empower (RISE 2.0) (6 months)	N = 41 women physicians, faculty members and advanced practice practitioners	Pre; Post-session; Post-short (1 month; 3 months)
Kelly, 2021, USA	Mixed-methods survey and interview study	Academic healthcare	Women’s Wellness through Equity and Leadership (WEL) (18 months)	N = 18 programme participants; n = 13 provided demographic survey data; early- to mid-career women physicians from multiple specialties	Post-imm after each series and at programme completion
Knipfer, 2017, Germany	Quantitative pilot pre–post evaluation	Academic STEMM	Not reported (8 months)	N = 16 women scientists, including postdoctoral researchers and junior group leaders, from three German research organisations	Pre/per-module; Post-imm; Post-short (2 mo)
Kvach, 2017, USA and Ethiopia	Qualitative descriptive study	Academic medicine	Not reported (2-week intensive fellowship, with mentoring encouraged for at least 1 year)	N = 8 women medical faculty from Ethiopia	Post-imm
Lämsä, 2019, Finland	Qualitative study	Professional and private sector	Female Forum (MBA Leadership Module) (2.5 years)	N = 22 mid-career professionals with extensive leadership and work experience	Pre; Post-long (18 months)
Lee, 2024, USA	Mixed-methods pre–post evaluation	Academic medicine	Early Career Women’s Leadership Program (ECWLP) (6 months)	N = 51 early-career women physicians and scientists participating in an academic medicine leadership programme	Pre; Post-imm
Martínez-Martínez, 2021, Spain	Mixed-methods case study	Corporate, multiple sectors	The Promociona Project (duration not reported)	N = 32 women executives from diverse industries who had recently been promoted	Post-long (retrospective, 1–6 years)
Mbilishaka, 2023, USA	Qualitative autoethnography	Academic higher education	Dialogues in Leadership Herstory (DiLH) (10 weeks)	N = 3 Black women Assistant Professors in psychology	Retrospective post-only
Megheirkouni, 2017, United Kingdom	Qualitative semi-structured interview study	Sport management	The Women Leadership Development Programme (3 years)	N = 10 women in middle or senior leadership roles across sports organisations	Retrospective post-only
Miles-Cohen, 2020, USA	Mixed-methods archival review	Psychology	Leadership Institute for Women in Psychology (5 days across two periods)	N = 339 mid-career women psychologists across 10 programmes	Mid; Post-imm; Post-long (retrospective)
Mohanakumar, 2022, Asia–Pacific region	Reflective descriptive account	Public administration: customs	Container Control Programme (4 weeks)	N = 58 women customs officers from 11 customs administrations	Post-imm
Nakamura, 2022, USA	Qualitative case study	Professional, multiple sectors	Not reported (3 days)	N = 7 women managers from multiple industries and countries	Mid; Post-imm; archival pre-programme material
Nash, 2018, Antarctica; Australia-run	Qualitative case study	Academic STEMM	Homeward Bound (3 weeks)	N = 25 women working in STEMM across five industrialised countries	Mid; Post-short (<2 months)
Nash, 2021, Antarctica; Australia-run	Qualitative longitudinal study	Academic STEMM	Homeward Bound (12 months, culminating in a 3-week Antarctic voyage)	N = 25 women working in STEMM across five industrialised countries	Mid; Post-long (1 year)
Nowling, 2018, USA	Quantitative programme evaluation	Academic medicine	Focused Career Development Program for Women Faculty (2-day programme; later half-day promotion-focused programme)	Approximately 200 attendees; approximately 160 included in promotion analyses; women faculty, primarily Instructors or Assistant Professors	Pre; Post-imm; Post-long (1–4 years)
O’Brien, 2022, United Kingdom	Qualitative grounded theory study	Outdoor education	Women’s Outdoor Leadership Course (Outward Bound) (10 weeks)	N = 8 early-career women programme participants	Post-imm
Parker, 2018, Australia	Mixed-methods survey evaluation	Academic higher education	Career Progression Program (12 months)	N = 51 women academics at Level C	Retrospective cross-sectional/post-only (programme cohorts 2010–2016)
Pelfrey, 2022, USA	Qualitative thematic interview study	Academic medicine	Future Leaders Exchange Program (FLEX) (7 months)	N = 25 FLEX graduates interviewed 1–8 years after programme completion	Pre; Mid; Post-imm; Post-long (retrospective, 1–8 years)
Peterson, 2019, Sweden	Qualitative case study	Academic higher education	Identity, Development, Advancement and Support (IDAS) (ongoing seminars and activities; total duration not reported)	N = 15 women university rectors from multiple institutions	Post-long (retrospective alumni study)
Round, 2024, Japan and Australia	Quantitative pre–post programme evaluation	Professional, multiple sectors	Japan–Australia Emerging Female Leader Program (6 months)	Approximately N = 170 women leaders from multiple countries and industries	Pre; Post-imm
Russell, 2023, USA	Mixed-methods evaluation	Academic and professional: family and consumer sciences	AAFCS Leadership Academy (duration not reported)	N = 25 AAFCS Leadership Academy participants/graduates	Retrospective post-only
Ryan, 2021, Ireland	Qualitative longitudinal study	Healthcare	Not reported (18 months)	N = 25 women leaders from six clinical sites	Mid; Post-imm
Schwartz, 2021, USA	Mixed-methods cross-sectional survey evaluation	Academic medicine	Women’s Empowerment and Leadership Initiative (WELI) (ongoing; evaluated at 2 years)	N = 92 women paediatric anaesthesiologists participating as protégés, advisors or dual protégé-advisors	Retrospective cross-sectional (ongoing programme; survey at ∼28 months after programme launch)
Sears, 2019, USA	Mixed-methods descriptive evaluation	Healthcare	Women Leaders in Medicine (WLiM) (half-day to full-day activities; total programme duration not reported)	More than 200 women physicians participated; 242 attended at least one programme activity	Pre-development; Post-imm; Post-short (6 months)
Sears, 2025, USA	Quantitative controlled cohort study	Healthcare	Women Leaders in Medicine (WLiM) (2 years)	N = 190 women physicians working in a multispecialty healthcare system	Pre; Mid; Post-imm
Selzer, 2017, USA	Qualitative collaborative autoethnography	Academic higher education	Women’s Leadership Program (7 months)	N = 3 women academics with doctoral qualifications and several years of university employment	Post-imm (reflective autoethnography)
Sims, 2022, USA	Mixed-methods follow-up study	Academic higher education	Women Faculty in Leadership Roles (WFLR) Program (9 months)	N = 28 tenured faculty members	Pre; Post-imm
Skarupski, 2019, USA	Quantitative retrospective cross-sectional study	Academic medicine	Leadership Program for Women Faculty (LPWF) (9 months)	Survey respondents n = 114 of 285 programme graduates; women faculty at Assistant Professor rank or higher	Post-long (retrospective survey, 1–8 years; retrospective pre-post ratings)
Spalluto, 2017, USA	Quantitative pre–post survey evaluation	Academic medicine	Leadership Intervention to Further the Training of Female Faculty (LIFT-OFF) (2 years)	N = 39 women radiology faculty and trainees	Pre; Mid
Sutton, 2024, USA	Prospective longitudinal single-cohort evaluation with paired pre-post measurements	STEMM	Sallie Rosen Kaplan Postdoctoral Fellowship for Women Scientists (10 months)	N = 111 postdoctoral women scientists across ten cohorts	Pre; Post-imm
Valerio, 2024, Asia–Pacific region	Qualitative case study	Academic STEM: agricultural research	Meryl Williams Fellowship (Women in Agricultural Research) (two-week and four-day immersive workshops)	N = 15 women agricultural researchers working primarily in mid-level roles and government research institutions	Post-imm
Van Oosten, 2017, USA	Perspective and descriptive programme account with illustrative participant outcomes	Academic STEM and corporate/ industry STEM	The Leadership Lab for Women (3 months)	More than 50 women working in STEM and manufacturing across five cohorts	Post-imm
Walsh, 2022, USA and LMICs	Uncontrolled post-intervention programme evaluation	Academic medicine and global health	Female Global Scholars Program (2 years)	N = 10 early-career women global health researchers from six countries	Pre; Post-imm

Note: AAMC, Association of American Medical Colleges; ELAM, Executive Leadership in Academic Medicine; ELATES, Executive Leadership in Academic Technology, Engineering and Science; EWIM, Early-Career Women in Medicine; LMICs, low- and middle-income countries; MWIM, Mid-Career Women in Medicine; N, number of participants; Post-imm, immediate post-programme assessment; Post-long, long-term follow-up of at least 1 year; Post-session, assessment after individual programme sessions or modules; Post-short, follow-up occurring more than immediately after programme completion but less than 1 year; Pre, assessment conducted before programme commencement; Mid, assessment conducted during the programme; STEM, science, technology, engineering and mathematics; STEMM, science, technology, engineering, mathematics and medicine; AAFCS, American Association of Family and Consumer Sciences.

The primary outcome was the level at which WLDP effectiveness was evaluated, operationalised using an extended Kirkpatrick framework that distinguishes subjective (self-reported) from objective (externally verifiable) indicators across seven levels (Table 2), a widely applied model for assessing training and development interventions.^40^ Synthesis followed the prescribed steps of narrative synthesis and involved framework-based categorisation, contextual mapping, and methodological sensitivity analysis.^31^ Six sensitivity and exploratory analyses examined whether the distribution of reported outcomes across extended Kirkpatrick levels differed: (i) between studies with and without a pre-programme baseline; (ii) between studies with and without a non-participant comparison group; (iii) between studies rated as having low vs. moderate or high risk of bias; (iv) between studies with and without long-term follow-up of at least 1 year; (v) between studies with and without reported organisational embedding or support; and (vi) in analyses restricted to pre-post studies and controlled studies (Supplementary Table S7A–F).

**Table 2:** Kirkpatrick’s extended evaluation levels and descriptions.

Level	Label	Description
Level 1	Reaction	Participants’ immediate satisfaction and perceived relevance/psychological safety (e.g., usefulness of content, pacing, accessibility, safe environment).
Level 2A	Knowledge (subjective)	Self-reported gains in principles, facts, attitudes, and skills by programme completion (e.g., confidence, leadership self-efficacy).
Level 2B	Knowledge (objective)	Demonstrated learning measured by objective means (e.g., assessment scores, pre- and post-assessments).
Level 3A	Behaviour/expertise (subjective)	Operationalised as transfer of learning to work, captured via self-, peer-, or manager-reported evidence (e.g., survey accounts of applying programme skills, speaking up in executive meetings, observation notes).
Level 3B	Behaviour/expertise (objective)	Tangible, observable behaviour change externally verifiable through documentation independent of participant self-report (e.g., number of stretch projects led, human-resources records of leadership appointments without promotion, records of grants and awards).
Level 4A	System results/performance (subjective)	Employee perceptions of an equitable, supportive culture (e.g., fairness of promotions, inclusion climate, flexibility without stigma).
Level 4B	System results/performance (objective)	Tangible organisational outcomes from administrative data (e.g., promotion velocity, pay-equity movement, increased retention).

Note. The original four-level model categorised outcomes as: reaction (participant satisfaction), learning (knowledge/skills), behaviour (application in practice), and results (organisational impact). Following prior adaptations of this framework,^32^^,^^39^ we applied an extended Kirkpatrick framework that distinguishes subjective indicators (e.g., self-reported perceptions or experiences) from objective indicators (externally verifiable indicators).

No effect estimates or heterogeneity statistics were calculated, and no statistical software was used; extracted outcomes were synthesised as reported without transformation or imputation. Risk of bias was assessed independently by two reviewers using design-appropriate Joanna Briggs Institute (JBI) Critical Appraisal Tools,^41^ with discrepancies resolved by a third reviewer and rated high (≤49% of 'yes' scores), moderate (50–69%), or low (≥70%; Supplementary Table S8A–F). Reporting bias was not formally assessed and GRADE was not applied because the review synthesised heterogeneous evaluations narratively rather than estimating certainty in a pooled effect; methodological quality and strength of inference were assessed instead through JBI appraisal and sensitivity analyses.

### Statistical analysis

Findings were summarised descriptively using counts and percentages. No inferential statistical analyses or meta-analysis were performed.

### Role of the funding source

The funder of the study had no role in study design, data collection, data analysis, data interpretation, or writing of the report.

## Results

**Fig. 1:**
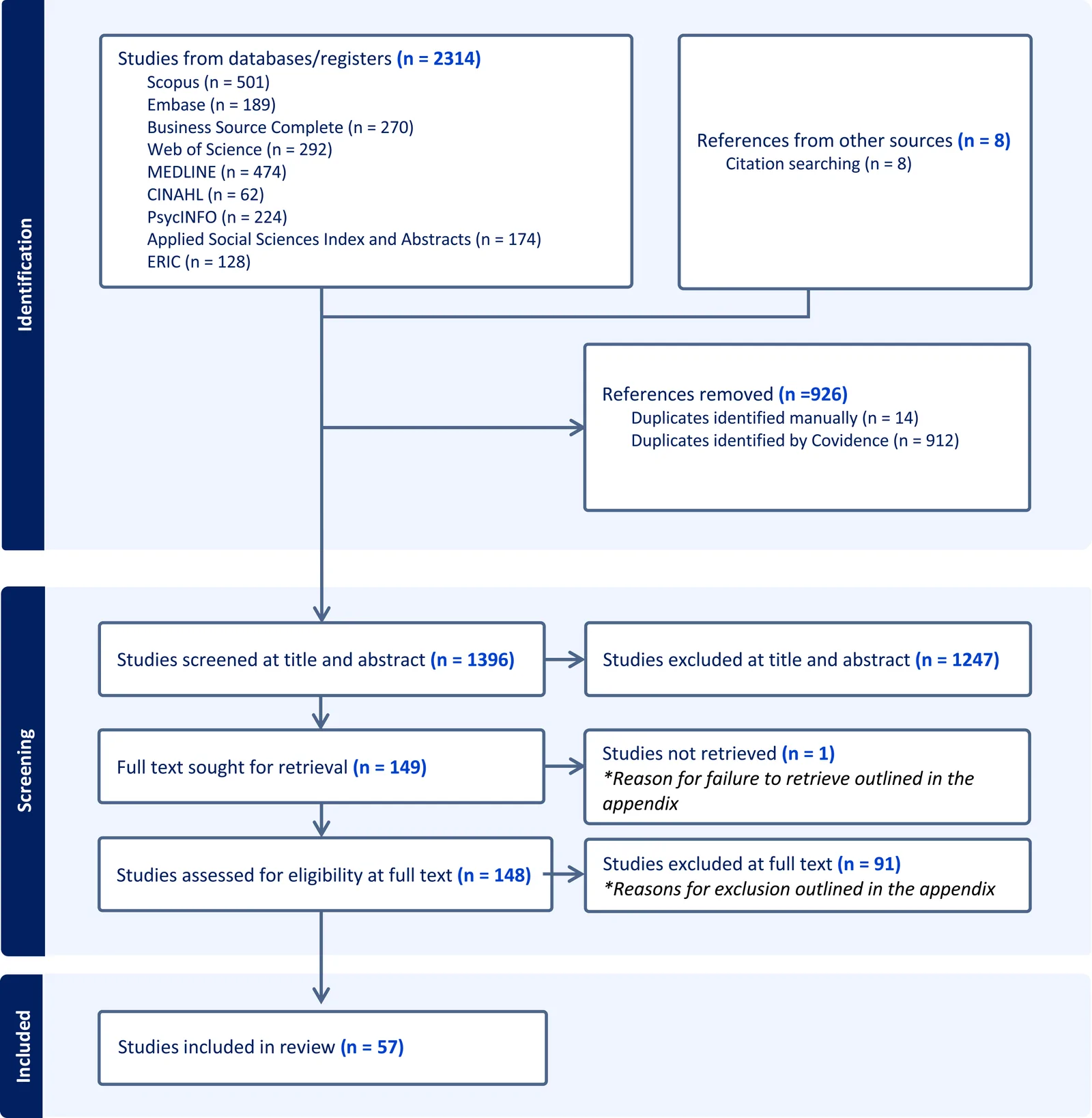
Preferred Reporting Items for Systematic Review and Meta-Analysis (PRISMA) flow chart.

From 2314 initial records, 57 studies were retained for synthesis (PRISMA flow diagram, Fig. 1; full list of included studies, Supplementary Table S9). Inter-rater agreement was substantial to almost perfect (title/abstract: κ = 0.68; full text: κ = 0.95; quality appraisal κ = 0.78). Reasons for exclusion at full-text and studies not retrieved are listed in Supplementary Table S10. Study designs, classified by the JBI appraisal tool applied (see Supplementary Table S8A–F), comprised qualitative studies (n = 20), mixed-methods studies (n = 19), quasi-experimental (pre-post) studies (n = 9), cohort studies (n = 5), analytical cross-sectional studies (n = 2), and descriptive programme reports appraised as text/opinion (n = 2). No study used randomisation; four studies (7%) included a comparison group.^42^, ^43^, ^44^, ^45^ Thirty-two studies (56%) were rated low risk of bias, 16 studies (28%) moderate, and nine studies (16%) high risk within their respective JBI design-specific tools. Strategies to address confounding were reported in four of 18 studies with applicable analytical designs (22%). The majority of papers used self-reported data (55/57, 96%) via surveys or questionnaires (24/55, 44%), interviews (12/55, 22%), the combined use of both (15/57, 26%), and other self-report methods (4/55, 7%; Supplementary Tables S3, S7D, S8C–F) S3, S7D.

The 57 included studies evaluated 52 unique WLDPs across 23 countries, concentrated in high-income countries (51/57 studies, 89%) mainly the United States (36/57 studies, 63%). Programmes were concentrated in academic sectors or disciplinary fields, accounting for 40 of 57 studies (70%), including academic health and medicine (n = 25, 44%) and other academic fields, such as higher education, STEMM, nursing, psychology, and agricultural research (n = 15, 26%). The remaining programmes operated in non-academic or cross-sector fields (17/57 studies, 30%; see Table 1). Organisational settings were predominantly universities and academic health institutions (33/57 studies, 58%), professional associations (6/57 studies, 11%), and government or public-sector organisations (5/57 studies, 9%); remaining settings detailed in Supplementary Table S3. Twenty-one studies (37%) evaluated programmes integrated within broader equity initiatives.

Delivery mode was explicitly reported in 24 studies (42%); in a further 32 studies (56%) the likely mode could be inferred from described programme activities, while it was unclear in one study. Modes included in-person, online or virtual, hybrid, and residential formats, with some reporting flexible delivery.^38^^,^^46^, ^47^, ^48^ Sample sizes ranged from 3 to 3268 (M = 186, Mdn = 35). Programme duration ranged from half a day to three years (12 months most common, n = 8). A pre-programme baseline was available in 23 of 57 studies (40%), while 24 studies (42%) had pre-post outcome measurement or a verified non-participant comparison group. Long-term follow-up of at least 1 year was reported in 16 studies (28%), with follow-up extending to 10 years. Twelve studies used institutional human-resources, administrative, archival, or programme records, nine studies used named validated or established psychometric instruments, and two used 360-degree feedback to assess outcomes or triangulate and contextualise evaluation findings. Study and programme characteristics are summarised in Table 1, with full extraction details provided in Supplementary Table S3 and TIDieR mapping in Supplementary Table S4. The complete list of included studies is provided in Supplementary Table S9.

Where recruitment was described (43/57 studies, 75%), 37 studies (86%) reported selective entry: 21 used nomination-based or invitation-only models, 15 competitive selection and one used a mixed selective model.^49^ Selection criteria and processes included supervisor or senior leader endorsement, institutional support, and existing leadership potential (Supplementary Table S5). Six programmes reported open enrolment accessible to all eligible women,^42^^,^^46^^,^^50^, ^51^, ^52^, ^53^ while one broadened access by transitioning from nomination-based entry to an open call.^51^ Six programmes explicitly incorporated equity-oriented recruitment beyond gender, prioritising diversity, underrepresented groups, or structural disadvantage.^24^^,^^28^^,^^29^^,^^37^^,^^54^^,^^55^ Participation support was described in 45 studies (79%) and included employer-paid fees,^42^^,^^51^^,^^56^ scholarships,^57^ stipends, travel assistance,^54^ and protected time.^29^^,^^55^^,^^56^

Demographic and other equity-relevant characteristics were inconsistently reported. Race or ethnicity was reported in 22 studies (39%); of these, 18 (82%) described predominantly White cohorts. Eight programmes described equity-oriented recruitment and subsequent cohort diversity.^28^^,^^29^^,^^37^^,^^38^^,^^54^^,^^55^^,^^57^^,^^58^ Seven programmes reported sexual orientation,^38^^,^^42^^,^^51^^,^^55^^,^^57^^,^^59^^,^^60^ two captured disability status,^42^^,^^57^ and one disaggregated advancement outcomes by race or ethnicity.^42^ No study reported across all PROGRESS-Plus dimensions (summary in Table 3; full study-level data mapped in Supplementary Table S5).

**Table 3:** Equity-relevant reporting and programme access across included studies.

PROGRESS-Plus domain	Studies reporting, n/57 (%)	Main finding
Place of residence	57 (100%)	All studies reported the country or countries associated with programme delivery or participant recruitment. More detailed information about participants' geographic location, such as rural, urban or regional residence, was reported in only 3/57 studies (5%).
Race, ethnicity, culture or language	22 (39%)	Reporting was inconsistent. Several cohorts were predominantly White, while some programmes enrolled racially and ethnically diverse participants. Language was rarely reported separately.
Occupation	57 (100%)	All studies described participants' broad occupational group. Participants were concentrated in academic medicine, higher education, healthcare, STEMM, management and other professional roles. Employment status, career stage and working conditions were reported less consistently.
Gender or sex	57 (100%)	All programmes primarily or exclusively targeted women. A small number included mixed-gender cohorts or referred to gender-diverse participants, but evaluation findings generally focused on women.
Religion	1 (2%)	Only one study referred to possible religious diversity, and no study systematically reported participants’ religion or faith affiliation.
Education	32 (56%)	Where reported or clearly identifiable from professional qualifications, participants were generally highly educated and commonly held medical, doctoral or other postgraduate qualifications.
Socioeconomic status, including proxy indicators	27 (47%)	Direct socioeconomic measures were rare. Proxy indicators included unemployment or career transition, occupational class, full-time or part-time employment, insecure contracts, career interruption, business ownership, participant fees, scholarships, stipends, protected time and employer-funded participation.
Social capital, including proxy indicators	38 (67%)	No study formally measured social capital. 20 studies reported a plausible social-capital proxy, such as senior leader nomination, targeted invitation, supervisor endorsement, organisational sponsorship, an internal sponsor, or association-based access; 18 reported selection/access information without a clear social-capital indicator, such as competitive selection, interview selection, application processes, or prior leadership experience; and 19 studies did not report any social capital indicators.
Plus: Personal characteristics associated with discrimination	Age: 26 (46%); sexual orientation: 7 (12%); disability: 2 (4%)	Age was most commonly reported, usually as a mean, range or grouped category. Sexual orientation and disability were rarely reported. These characteristics were seldom examined in relation to programme access or outcomes.
Plus: Relationship and family characteristics	Children or caring responsibilities: 13 (22%); marital or relationship status: 9 (16%)	Reported characteristics included marital or partner status, children, pregnancy, childcare, elder care and other caring responsibilities. These factors were seldom assessed in relation to programme access, completion or outcomes.
Programme accessibility and participation support	45 (79%)	Participation support (45/57 of studies) and recruitment (43/57 of studies) was described in most studies. Entry was commonly selective or targeted through nomination, invitation, application, employer endorsement or competitive selection. Reported supports included employer-paid fees, scholarships, stipends, travel assistance, protected time, release from duties and use of professional development time.
Programme funding	40 (70%)	Funding was most commonly provided by universities, employers, professional associations, health systems, government grants, philanthropic donors or corporate sponsors. Seventeen studies did not clearly report programme funding.
Integration within a broader equity initiative	21 (37%)	Most programmes operated as stand-alone leadership development initiatives rather than as components of broader organisational equity strategies.

Note. PROGRESS refers to place of residence; race, ethnicity, culture and language; occupation; gender or sex; religion; education; socioeconomic status; and social capital. Plus encompasses personal characteristics associated with discrimination and relationship or family characteristics that may contribute to disadvantage. Socioeconomic status and social capital include direct reporting and broader proxy indicators. Socioeconomic proxies included employment position and security, occupational class, career interruption and access to financial or workplace resources. Social-capital proxies included professional networks, mentoring, sponsorship, senior leader endorsement and organisational support. Counts indicate explicit reporting of the characteristic or proxy within the included study reports. Categories are not mutually exclusive. STEMM, science, technology, engineering, mathematics and medicine.

Programme rationales frequently attributed women’s underrepresentation to structural and institutional factors, citing gender inequality,^54^^,^^55^^,^^61^^,^^62^ the leaky pipeline,^59^^,^^60^^,^^63^^,^^64^ gender bias,^55^^,^^65^ gender gap,^35^^,^^36^^,^^66^ and SDG5.^63^ Programme components in all 57 studies were directed at individual participants and commonly included skills training, workshops, coaching, mentoring, networking, and leadership identity development. Only one programme reported organisation-facing components, including sexual-harassment policy development and gender-office activities.^61^ Forty studies (70%) reported at least one form of organisational support, including senior-leadership sponsorship,^37^^,^^67^ protected time,^29^^,^^55^^,^^68^ departmental culture initiatives,^50^ and infrastructure intended to support gender equity.^55^ A theory of change or relevant theoretical framework, defined as an explanation of how or why the programme was expected to produce change, was identified in 23 studies (40%). A theoretical or pedagogical model guiding programme content or learning activities was reported in seven studies (12%). A named or identifiable framework guiding evaluation or outcome classification was reported in 16 studies (28%), most commonly Kirkpatrick (n = 8). Thirty-nine studies (68%) used summative evaluation only. Explicit alignment, defined as the traceable link between the stated rationale, the programme components delivered, and the outcomes measured was identified in four studies (7%).^37^^,^^55^^,^^64^^,^^69^ See Supplementary Table S3 for more detail on programme aims, intervention design, and evaluation.

**Fig. 2:**
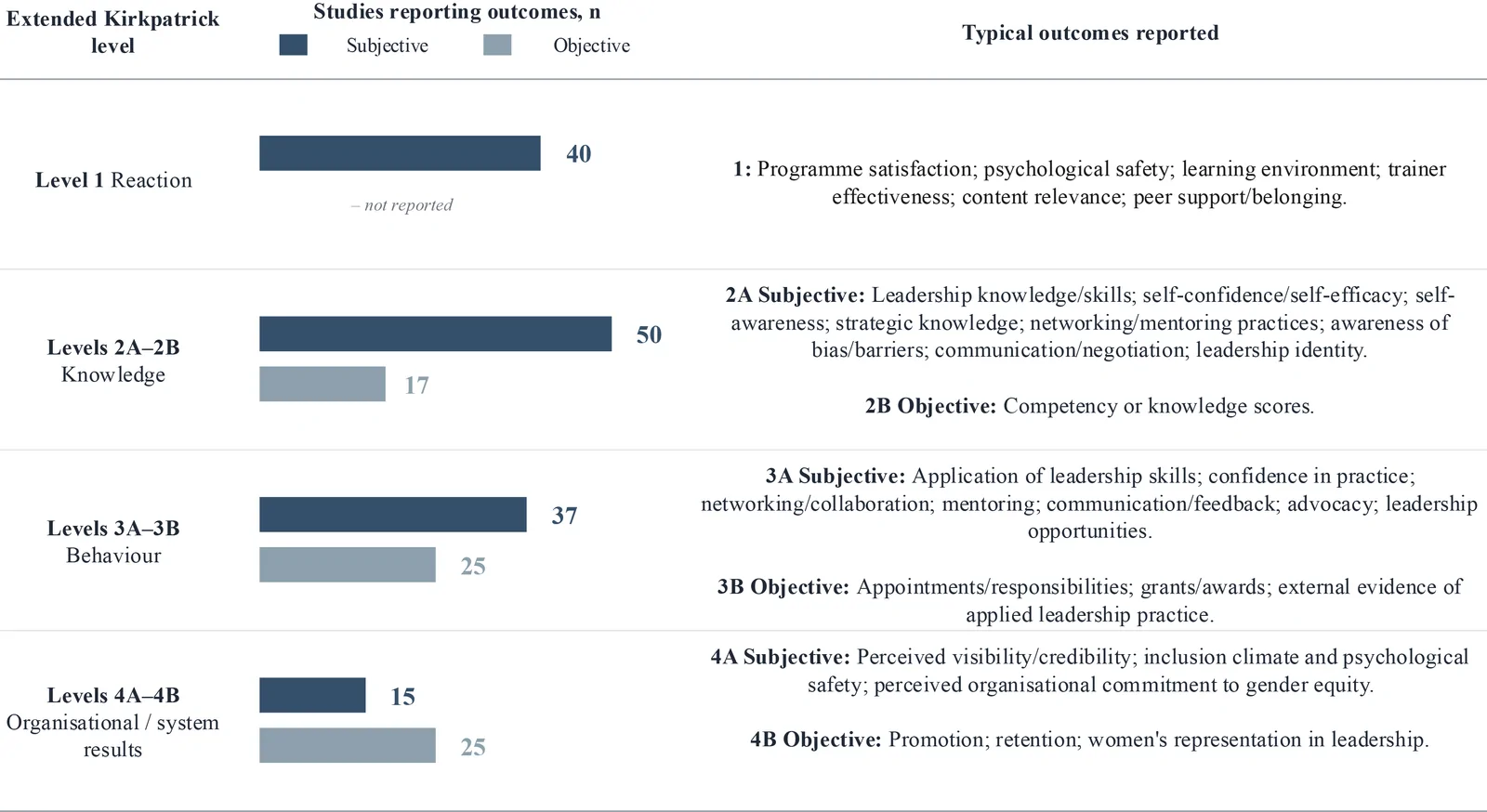
Bars show the number of the 57 included studies reporting outcomes at each level, drawn to a common scale. Within each level, subjective indicators (A) capture self-reported or perceived outcomes and objective indicators (B) capture externally verifiable measures. Categories are not mutually exclusive: a study could report outcomes at more than one level and could report both subjective and objective indicators, so counts exceed 57 and do not sum to 100%. Level 1 (Reaction) comprises subjective indicators only; no objective Level 1 outcomes were reported (−). Typical outcomes reported summarises the indicator types coded at each level and is illustrative, not exhaustive.

Outcomes were coded according to the extended Kirkpatrick framework and are presented in Fig. 2 and Supplementary Table S6. Individual-level outcomes (Levels 1–3) were reported in all 57 studies (100%); any organisational outcome (Level 4) was reported in 32 (56%). Subjective outcomes were reported by a greater proportion of studies than objective outcomes at Level 2 (50/57, 88% vs. 17/57, 30%) and Level 3 (37/57, 65% vs. 25/57, 44%), whereas this pattern reversed at Level 4 (15/57, 26% subjective vs. 25/57, 44% objective). Reaction outcomes (Level 1) were reported in 40 studies (70%). Subjective knowledge outcomes (Level 2A) were reported in 50 (88%), and objective knowledge (Level 2B) in 17 (30%), assessed via pre-post testing. Subjective behaviour outcomes (Level 3A) were reported in 37 (65%), drawn from participant or colleague accounts of applying programme learning at work. Objective behaviour outcomes (Level 3B) were reported in 25 (44%), evidenced by externally verifiable records such as documented leadership appointments,^29^^,^^57^^,^^69^ grants or awards.^57^^,^^63^^,^^69^ Subjective organisational outcomes (Level 4A) were reported in 15 (26%), and objective organisational outcomes (Level 4B) in 25 (44%), drawn from institutional promotion and retention data.^44^^,^^45^^,^^69^^,^^70^ Four studies (7%) included a verified non-participant comparison group, three of which reported objective organisational outcomes (Supplementary Tables S7B and S6).

Studies with a pre-programme baseline (23/57, 40%) more frequently reported objective knowledge outcomes than studies without one (Level 2B: 16/23, 70% vs. 1/34, 3%), while studies without a baseline more frequently reported subjective knowledge outcomes (Level 2A: 32/34, 94% vs. 18/23, 78%). Reporting of any Level 4 outcome was similar between groups (13/23, 57% vs. 19/34, 56%; Supplementary Table S7A). All four studies with a verified non-participant comparison group reported a Level 4 outcome, three of them objective (Supplementary Table S7B). Among studies at low risk of bias, 16/32 (50%) reported any Level 4 outcome, compared with 32/57 (56%) overall. Objective knowledge outcomes were less frequently reported in low-risk studies than in those at moderate or high risk (3/32, 9% vs. 14/25, 56%), partly reflecting the concentration of single-group pre-post and administrative outcome designs in the moderate- or high-risk group (Supplementary Table S7C).

Studies with follow-up of at least 1 year (16/57, 28%) more frequently reported objective behavioural outcomes (Level 3B: 10/16, 63% vs. 15/41, 37%), any Level 4 outcome (13/16, 81% vs. 19/41, 46%), and any objective Level 3B or 4B outcome (14/16, 88% vs. 19/41, 46%) than studies without long-term follow-up (Supplementary Table S7D). In exploratory analyses, studies reporting organisational embedding or support more frequently reported any Level 4 outcome (25/40, 63% vs. 7/17, 41%) and objective organisational outcomes (19/40, 48% vs. 6/17, 35%). All studies using administrative or externally verifiable records, and all four with a comparison group, fell within this category (Supplementary Table S7E). Restricting the synthesis to the 24 studies with pre-post measurement or a verified non-participant comparison group, objective knowledge outcomes were reported in 16/24 (67%) compared with 17/57 (30%) overall, and objective organisational outcomes in 13/24 (54%) compared with 25/57 (44%); reporting of any Level 4 outcome was similar (14/24, 58% vs. 32/57, 56%; Supplementary Table S7F).

## Discussion

This review identified two recurring patterns in how WLDPs are accessed and evaluated. First, an equity paradox in reported access: programmes aimed at reducing gender inequality frequently used selective recruitment processes reaching women who were already institutionally visible. Second, an implementation-evaluation gap: programmes framed as equity strategies were predominantly delivered to, and evaluated at, the level of the individual. These findings extend previous reviews of effective organisational interventions for advancing women in healthcare leadership, which emphasise integrated approaches spanning institutional commitment, stakeholder engagement, policy reform, mentoring and leadership development, evaluation and reporting.^21^ They are also consistent with qualitative reviews showing that participants value WLDPs for confidence, networks, leadership identity, and career support.^27^ This review adds that the dominant evaluation logic remains poorly matched to the equity claims made for these programmes: individual-level benefits are well documented, but organisational change, equitable access, and attribution remain insufficiently assessed.

The equity paradox runs through the evidence base. Where recruitment was described (43 studies), selective entry accounted for 37 (86%), most requiring supervisor endorsement or institutional visibility, processes that tend to reach women already recognised as ‘high-potential’ and embedded in sponsorship networks (Table 3).^18^^,^^58^^,^^71^ Selective recruitment may reflect programme aims and organisational context, and where the intended outcome is organisational change, targeting women with the access and resources needed to effect it is defensible. Whether this produces stronger organisational outcomes, however, was not established in the evidence reviewed. Aggregate 'women's advancement' metrics, disaggregated by race or ethnicity in only one included study,^42^ cannot show whether gains distribute equitably or concentrate among the already-recognised, and pathways into programmes independent of sponsorship were rarely described. Treating leadership development solely as a resource for high-potential individuals also risks reproducing the meritocratic logic that obscures how race, class, and disability shape who is seen as having ‘potential’.^25^^,^^72^ Progress towards SDG5’s goal to empower *all* women requires reconceptualising WLDPs as equity interventions with transparent selection criteria, accessible pathways, and disaggregated outcomes; without these, WLDPs may concentrate development opportunities among women who already have institutional recognition rather than addressing inequities in who can enter and benefit from leadership development.^14^^,^^15^^,^^53^^,^^73^

Limited organisational evidence is not evidence of ineffectiveness. Organisational change of the kind WLDPs are positioned to contribute to unfolds over years and depends on an enabling environment. Individual leadership development alone cannot produce organisational transformation; programmes intended to advance gender equity must also address the structures, cultures, resources, and opportunity pathways that constrain women’s advancement.^22^^,^^74^ Capturing such change demands long follow-up, administrative data infrastructure, and dedicated evaluation funding that few programmes command; WLDPs are typically resourced for delivery rather than for robust longitudinal evaluation. Consistent with this, organisational outcomes were associated with institutional investment — executive sponsorship, protected time, dedicated funding, and equity-oriented infrastructure — rather than with distinctive programme content, and were concentrated in mature programmes and in sectors, notably academic medicine, where equity commitments were institutionalised and data systems enabled tracking of appointments, promotions, and retention.^29^^,^^42^^,^^69^^,^^70^ Even among these, organisational evidence relied predominantly on internally generated or self-perceived indicators, with few evaluations using independent assessors or follow-up sufficient to confirm systemic change. Organisational outcome reporting did not materially increase among studies at low risk of bias or when the synthesis was restricted to pre-post or controlled designs (Supplementary Table S7C and F), indicating that the scarcity of organisational evidence reflects what programmes measure rather than the inclusion of weaker studies.

These patterns also raise questions about how WLDP impact is defined and by whom. Feminist and participatory evaluation traditions recognise lived experience as a legitimate source of knowledge, and the field’s reliance on qualitative and subjective designs may reflect methodological fit for small, context-specific programmes rather than weakness alone.^22^^,^^75^^,^^76^ However, changes in confidence, voice, belonging, or leader identity do not demonstrate that the organisational and systemic barriers participants face have changed. We therefore use the extended Kirkpatrick framework to show what is and is not measured, not to position objective organisational outcomes as the only valid evidence of value; a stronger evidence base would integrate rich accounts of individual change with longitudinal, objective organisational indicators.

The implementation-evaluation gap reflects a misalignment between how programmes are conceptualised, justified, and how they are designed and assessed. Programmes framed in structural terms — referencing gender inequality,^59^^,^^61^^,^^77^ the leaky pipeline,^51^^,^^78^^,^^79^ gender bias,^65^^,^^80^ gender imbalance,^62^ and SDG5^81^ — were conceived, delivered, and evaluated as individual professional development interventions. This places the burden of change on women navigating inequitable systems rather than on organisations and the systems themselves.^82^^,^^83^ The implication is shared responsibility: accountability for translating individual gains into systemic change cannot rest with programmes or participants alone but must extend to the institutions that commission them. For WLDPs to meaningfully advance SDG5, investment in WLDPs and their evaluation must move, where feasible, toward objective, longitudinal, and transparent equity metrics tied to organisational accountability, capable of rendering progress, or stagnation, empirically visible.^22^^,^^84^ This is particularly urgent in healthcare and academic medicine, where WLDPs are concentrated and where gender disparities in leadership have a direct impact on healthcare quality and equity. This review directly informs the large-scale international NHMRC-funded initiative ‘Advancing Women in Healthcare and Academic Leadership’ and the National Women in the Health and Medical Sciences: Decadal Plan.^85^

The strengths of this review were a prospectively registered protocol, independent duplicate screening, extraction, and quality appraisal, a large and recent international evidence base, and an extended Kirkpatrick framework distinguishing individual from organisational and subjective from objective indicators. Several limitations constrain interpretation. Heterogeneity in study designs, WLDP definitions, and outcome measures precluded meta-analysis and complicated comparison. The absence of randomisation and the small number of comparison groups limit causal attribution, and the predominance of self-reported, immediate post-programme outcomes restricts assessment of sustained change. Geographic and demographic concentration limits generalisability to lower-resource and intersectionally diverse contexts. Because socioeconomic and intersectional characteristics were inconsistently reported, the extent of participant advantage could not be empirically verified; both the equity paradox and the implementation-evaluation gap should therefore be read as patterns in the available evidence rather than demonstrated properties of the programmes themselves. Exclusion of grey literature and non-English publications may have introduced publication and language bias, under-representing locally implemented, unpublished, or non-Western WLDP evaluations.

Findings of this review echo the 2025 UN gender snapshot’s conclusion that safeguarding gains in gender equality requires ambitious investments, coordinated action and better data.^86^ A persistent gap remains between the equity aspirations of WLDPs and their design, implementation, and evaluation. WLDPs consistently benefit their participants; the open question is whether, and under what organisational conditions, they contribute to shifting the inequitable patterns that produce gender leadership gaps. This requires reconceptualising WLDPs as integrated, contextually tailored approaches aligned to the structural conditions in which women lead, and a reorientation of accountability: from preparing women to navigate inequitable structures towards organisations sharing responsibility for dismantling them. In healthcare and academic medicine, this reorientation is both an equity imperative and a health-system priority, because leadership composition shapes workforce culture, research agendas, organisational decision-making, and the capacity of health systems to respond equitably to the populations they serve.

## Contributors

DM conceived the study design, led the systematic search, screened and extracted data, conducted quality assessment, performed the narrative synthesis, and drafted the manuscript. HT provided senior oversight and supervision, obtained funding, contributed to study conception, interpretation, drafting and critical revision of the manuscript. KR contributed to study design, interpretation of findings, and critical revision of the manuscript. SR contributed to data extraction, quality assessment, and critical revision of the manuscript. DM and SR accessed and verified the underlying data. All authors had full access to all data in the study and had final responsibility for the decision to submit for publication. All authors have read and approved the final manuscript.

## Data sharing statement

The study-level data extracted for this systematic review, including the characteristics, quality-appraisal assessments, and outcome classifications used in the synthesis, are provided in the Supplementary Appendix. The review protocol is available through PROSPERO under registration CRD42024554419. Additional extraction materials may be made available by the corresponding author upon reasonable request, subject to approval by the study team.

## Declaration of interests

HT reports support for the present manuscript from the Australian National Health and Medical Research Council (NHMRC) through Partnership Project grants APP1198561 and APP2018718 and NHMRC Fellowship APP2009326, with funding paid to her institution. HT leads the NHMRC-funded international initiative, ‘Advancing Women in Healthcare and Academic Leadership’, which draws on findings from this systematic review. HT reports no other relationships, activities, or interests relevant to the submitted work. All other authors declare no competing interests.
