# Supplementary material for: Women’s leadership development programmes and gender equality: a systematic review in healthcare, academia and beyond
Source: eClinicalMedicine. 2026 Sep 1;99:104185. doi: 10.1016/j.eclinm.2026.104185 (PMC13571131; doi:10.1016/j.eclinm.2026.104185)
Supplement: Supplementary file 1 [file mmc1.docx]

Supplementary Appendix: Tables S1-S10

Women’s Leadership Development Programmes and Gender Equality: A Systematic Review in Healthcare, Academia and Beyond

## Contents

Supplementary Table S1. SWiM (Synthesis Without Meta-analysis) reporting checklist

Supplementary Table S2. Ovid MEDLINE search strategy

Supplementary Table S3. Detailed characteristics of included women’s leadership development programmes

Supplementary Table S4. TIDieR mapping of extracted programme characteristics

Supplementary Table S5. Equity-relevant participant characteristics and programme accessibility mapped to PROGRESS-Plus

Supplementary Table S6. Study-level outcomes for women mapped to the extended Kirkpatrick framework

Supplementary Tables S7A-S7F. Sensitivity and exploratory analyses

Supplementary Tables S8A-S8F. Design-specific Joanna Briggs Institute critical appraisal results

Supplementary Table S9. Reference list of all included studies

Supplementary Table S10. Studies excluded at full-text screening and not retrieved, with reasons

# Supplementary Table S1. SWiM (Synthesis Without Meta-analysis) reporting checklist

| **SWiM item** | **Description** | **Location in manuscript** |
| --- | --- | --- |
| 1. Grouping studies for synthesis | Studies grouped by extended Kirkpatrick outcome level; sensitivity and exploratory groupings by presence of a pre-programme baseline, presence of a non-participant comparison group, risk of bias, long-term follow-up, and reported organisational embedding/support | Methods: Data extraction, synthesis and analysis; Table 1; Supplementary Tables S7A-S7F |
| 2. Standardised metric and transformation | No transformations undertaken; outcomes synthesised as reported | Methods: Data extraction, synthesis and analysis |
| 3. Synthesis method | Narrative synthesis informed by Popay et al., with framework-based categorisation using the extended Kirkpatrick framework | Methods: Data extraction, synthesis and analysis; reference 31 |
| 4. Criteria for prioritising results | Primary outcome prespecified as the level at which programme effectiveness was evaluated | Methods: Data extraction, synthesis and analysis |
| 5. Investigation of heterogeneity | Sensitivity and exploratory analyses by presence of a pre-programme baseline, presence of a non-participant comparison group, risk of bias, long-term follow-up, and reported organisational embedding/support | Methods; Findings: Sensitivity analyses; Supplementary Tables S7A-S7F |
| 6. Certainty of evidence | GRADE was not applied because of substantial heterogeneity in interventions, designs and outcomes; JBI critical appraisal and sensitivity analyses were used to assess the robustness of the evidence | Methods: Data extraction, synthesis and analysis |
| 7. Data presentation methods | Frequency counts and percentages by extended Kirkpatrick level, with tabular and graphical presentation of study-level and synthesised results | Findings; Figure 2; Table 3; Supplementary Table S6 |
| 8. Reporting results | Counts and denominators reported for the overall synthesis and each sensitivity or exploratory stratum | Findings; Supplementary Tables S7A-S7F |
| 9. Limitations of synthesis | Substantial clinical and methodological heterogeneity, absence of randomisation, limited use of comparison groups, sparse demographic reporting, and possible confounding by sector and programme context | Discussion: limitations |

# Supplementary Table S2. Ovid MEDLINE search strategy

1. ("leadership development program*"or"leadership development programme*"or"leadership training program*"or"leadership training programme*"or"leadership enhancement program*"or"leadership enhancement programme*"or"leadership development course*"or"leadership training course*"or"leadership enhancement course*"or"leadership initiative*"or"executive training"or"executive education"or"executive training program*"or"executive training programme*"or"leadership training initiative*"or"executive development workshop*"or"leadership skills program*"or"leadership skills programme*"or"leadership excellence program*"or"leadership excellence programme*"or"leadership advancement course*"or"executive education program*"or"executive education programme*"or"leadership mastery program*"or"leadership mastery programme*"or"leadership academ*"or"executive leadership series").ti,ab.

2. (leader* adj3 (develop* or train* or program* or programme* or course* or initiative* or academ* or workshop* or education or enhancement or advancement or excellence or skill*)).ti,ab.

3. (executive adj3 (train* or education or develop* or program* or programme* or workshop* or leadership)).ti,ab.

4. 1 or 2 or 3

5. (women or woman or female* or gender or"gender equity"or"gender equality"or"women leaders"or"female leaders"or"women in leadership").ti,ab.

6. exp Women/ or exp Female/ or exp Sex Factors/

7. 5 or 6

8. (outcome* or effect* or impact* or result* or benefit* or"return on investment"or"program evaluation"or"programme evaluation"or"program effectiveness"or"programme effectiveness"or"participant outcomes"or"organizational outcomes"or"organisational outcomes"or"career advancement"or"career development"or promotion* or retention or"skill development"or"skills development"or (behavio?r* adj1 change*) or"performance improvement"or"performance change"or evaluat* or effectiveness).ti,ab.

9. exp Program Evaluation/ or exp Outcome Assessment, Health Care/ or exp Career Mobility/ or exp Personnel Management/ or exp Organizational Innovation/

10. 8 or 9

11. 4 and 7 and 10

12. limit 11 to english language

13. limit 12 to yr="2015-Current"

14. limit 13 to journal article

# Supplementary Table S3. Detailed characteristics of included women’s leadership development programmes

| **Study** | **Sector/ field (organisational setting)** | **Programme aim/content** | **Theoretical/pedagogical model** | **Theory of change** | **Type of evaluation** | **Evaluation framework** | **Detailed data collection timepoints** | **Data sources and measures** |
| --- | --- | --- | --- | --- | --- | --- | --- | --- |
| **Adeola, 2024** | Academic nursing (Midwest Nursing Research Society, professional society) | The Leadership Academy’s aims are threefold: to expand the pool of MNRS members ready for effective leadership, to enhance leadership diversity within the society, and to foster member involvement in board leadership roles | Transformational leadership framework/style | Not reported | Formative and summative | Not reported | Post-imm: single participant survey administered at the close of the 11-month Leadership Academy cohort (2022-2023). | Closed post-programme survey (5-point Likert items covering knowledge gains, mentoring value and confidence, plus one open-ended prompt asking which session was most useful); descriptive summary only. |
| **Barnard, 2022** | Academic Higher Education (multi-institution, UK-wide programme) | “to be relevant to a broad base of employees in order to push for a critical mass of women in the sector” | Not reported | Not reported | Formative and summative | Not reported | Pre + Post-short + Post-long: online survey administered at four waves; T1 pre-Aurora (baseline), T2 at 3-6 months post-programme, T3 at 15-18 months post-programme, T4 at ≥3 years post-programme; interview and diary sub-sample followed longitudinally across T2-T4 (n=32 at T2, 19 at T3, 12 at T4). | Bespoke online questionnaires (demographics, career history, workplace culture and practice perceptions, engagement with leadership, self-rated leadership skills and styles, stress, work-life balance, non-work responsibilities, career strategies, and 12-14 Aurora-impact items rated increase/no effect/decrease); semi-structured interviews (leadership activities, enablers and hindrances, leadership styles encountered, structural and cultural barriers); reflective diaries; SPSS v23 for descriptive frequencies; NVivo deductive/inductive coding of qualitative data. |
| **Brixey, 2025** | Academic Medicine (university medical school) | aimed to address existing discrepancies in access to educational opportunities and promotional advancement between men and gender minorities (women and non-binary individuals) | Elements of experiential learning as illustrated in Kolb’s four stages of learning | Not reported | Formative and summative | Kern model | Pre; Post-imm: closed survey administered before the LAWFFT-UP series began and again immediately after the final of the eight events (delivered across 21 months). | Self-developed electronic pre- and post-intervention surveys (Qualtrics), informed by the earlier LIFT-OFF needs-assessment survey. The 72-item baseline survey assessed interest in promotion/leadership, professional-development gaps in teaching, research and service, and perceptions of gender inequity. The post-survey repeated relevant baseline items and added event attendance and perceived impact/knowledge-gain questions. |
| **Brue, 2016** | Academic STEM / career and technology education (state CareerTech system) | “Empower women to develop leadership strategies, provide a forum to share leadership insights, and address organisational challenges faced by women” | Not reported | Not reported | Summative | Not reported | Retrospective (post-only, timing not specified): single phenomenological interview with seven programme alumnae; interval since programme completion not reported. | Phenomenological semi-structured interviews with seven Oklahoma CareerTech alumnae (~90 minutes; open-ended prompts about lived programme experience); thematic phenomenological analysis focused on meaning and outcomes. |
| **Brue, 2018** | Academic STEM / career and technology education (state CareerTech system) | “facilitate women leaders by providing leadership knowledge, processing organisational challenges faced by women, and creating a safe space for women to share leadership insights | Not reported | Not reported | Summative | Not reported | Retrospective (post-only, timing not specified): same alumnae cohort as Brue 2016, re-analysed through a leader-identity lens; interval since programme not reported. | Same interview corpus as Brue 2016, re-coded for leader-identity construction; grounded-theory-informed thematic analysis emphasising role internalisation. |
| **Chang, 2016** | Academic medicine (national programmes; AAMC/Drexel ELAM) | EWIM & MWIM: “aim to inspire and provide academic career skills for early and mid-career faculty” ELAM: “to build capacity for individuals and institutions for leadership and increase the number of women faculty in leadership” | Not reported | Not reported | Summative | Not reported | Post-long (annual tracking ≤ 10 years): AAMC Faculty Roster HR records tracked annually for up to 10 years post-programme; matched-comparator retention and promotion analyses across CDP participants and non-participants. | Retrospective institutional HR data (5-15-year window) from the AAMC Faculty Roster; logistic regression comparing promotion and retention rates for CDP participants against matched non-participant controls; secondary institutional promotion/tenure dataset used for triangulation. |
| **Chang, 2020** | Academic medicine (national programmes) | EWIM and MWIM “faculty to increase their individual professional effectiveness in academic medicine” ELAM “programme for increasing both leadership capacity and the number of women faculty prepared for executive leadership” | Not reported | Not reported | Summative | Not reported | Pre; Post-long (5 years + 10 years): AAMC Faculty Roster records established participants’ academic rank, institution, degree and appointment history before or at programme participation; promotion to the next academic rank was then assessed overall and within 5- and 10-year intervals following the relevant initial appointment/programme period. | Retrospective AAMC Faculty Roster administrative data linking CDP attendance records with academic rank, appointment dates, degree, institution, tenure status and subsequent promotion. Promotion from Assistant to Associate Professor and Associate to Full Professor was compared between CDP participants and two comparison groups, non-participant women and men matched on institution, degree type, academic rank and year of appointment; analyses included Kaplan-Meier survival curves, log-rank tests and adjusted logistic regression for overall, 5-year and 10-year promotion outcomes. |
| **Chasserio, 2024** | Professional, cross-sector (French programme for women managers) | ‘focusing on developing self-confidence, self-efficacy, assertiveness, and leadership skills, through personal reflection, teamwork, and networking” | Not reported | transformative learning theory | Summative | Not reported | Pre; Post-short (6 mo): qualitative surveys administered prior to the 8-month programme and again 6 months after training; 13 semi-structured interviews were conducted with participants who had completed the programme at least 6 months earlier. Participant observation occurred during two training sessions and three networking events; 10 end-of-programme self-reports were also available. | Two-step qualitative open-ended surveys assessing self-confidence, self-efficacy, relationships with others, managing change, career progress and work-life balance; 47 participants completed both surveys. Additional data included 13 semi-structured interviews, participant observation, participant workbooks and 10 end-of-programme self-assessment reports. Data were analysed interpretively using thematic coding. |
| **Chaudron, 2021** | Academic medicine (university medical centre) | “to address topics that are crucial for faculty to have knowledge of, and skills in, to achieve leadership roles” | Not reported | Not reported | Summative | Not reported | Pre; Post-imm; Post-long (1-year follow-up): pre-programme self-assessment; end-of-programme self-assessment (Post-imm); email-based promotion/leadership update at ≈1 year post-programme. | Bespoke closed self-assessment survey (leadership competencies, self-efficacy, mentoring); email requests to alumnae for updated promotion status and leadership appointments at 1-year follow-up. |
| **Cordeiro, 2024** | Academic medicine and global health (US academic centre with LMIC partners) | “aims to promote the retention and advancement of early-career female investigators conducting global health research” | Not reported | Social Learning Theory and Intersectional Leadership Framework. | Summative | Not reported | Retrospective post-only: anonymous electronic survey completed between September and October 2021 by 12 of 22 current and former programme participants; semi-structured interviews conducted in May 2022 with 13 current participants. No prospective pre-programme survey was administered. | Anonymous REDCap survey collecting demographics, academic milestones, research outputs and Clinical Research Appraisal Inventory (CRAI)* self-efficacy scores; semi-structured interviews exploring mentorship, career development, gender bias, conflict resolution and curriculum feedback; descriptive quantitative analysis and thematic analysis. |
| **Danhauer, 2019** | Academic medicine (university school of medicine) | “Promote the careers of women faculty by enhancing their leadership skills.” | Not reported | Not reported | Formative and summative | Kirkpatrick | Pre + Post-imm: online survey (Likert + open-ended) administered before the first session and again at the end of the 6-month programme; logic-model outcomes captured across four cohorts (2014-2017). | Online surveys with Likert scales and open-ended items on most useful and least useful programme components; logic-model mapping of intended outcomes incorporating supervisor feedback and programme committee input to triangulate self-reported gains. |
| **David, 2025** | Academic medicine (Yale School of Medicine) | “The focus of the WLDP is on helping early-career women faculty build a support network with one another, enhance their leadership capacity, and gain skills and comfort navigating career challenges.” | Not reported | Not reported | Formative and summative | Logic model | Retrospective post-only: one anonymous Qualtrics survey administered in 2023-2024 to alumnae from eight cohorts conducted between 2020 and 2024. The study did not use a prospective baseline, pre-post design or longitudinal follow-up. | Author-developed retrospective Qualtrics survey containing demographic questions, 10 Likert items on programme satisfaction, leadership confidence, skills and post-programme leadership activity, and open-ended qualitative questions. CliftonStrengths and Everything DiSC Workplace were programme activities, not evaluation outcome measures. |
| **DeFrank-Cole, 2017** | Academic higher education (US public university) | empower women in leadership roles by providing them the tools, resources, development opportunities, and professional networks that will enable them to reach their full potential.” | Not reported | Self- Determination Theory | Summative | Not reported | Pre; Post-imm: group-administered pre-test at the Time 1 introductory meeting and post-test at Time 2 (end of group coaching, ≥4 months later); focus-group interviews conducted after the post-test in three programme phases. | Pre/post-test surveys with items rated 1 (“least effective”) to 4 ("most effective") across strategic thinking, influencing others, speaking up and network quality/quantity; paired-samples t-tests across three programme phases; alumni reflection surveys; separately convened post-programme focus groups (in three phases) with grounded-theory coding categorised against Self-Determination Theory dimensions (relatedness, competence, autonomy). |
| **Deutsch, 2022** | Academic medicine (Society for Pediatric Anesthesia, national professional society) | “to empower highly productive women pediatric anesthesiologists to achieve equity, promotion, and leadership” across six career development domains (promotion and leadership, networking, projects, mentoring, career satisfaction, well-being) | Not reported | Not reported | Formative and summative | Not reported | Mid/post-enrolment; repeated follow-up (6 mo; 14 mo). The first survey was administered after WELI had already commenced and therefore was not a true pre-programme baseline. | Emailed surveys (Likert items on impact across six career-development domains plus free-text on career, network and well-being impact); descriptive quantitative analysis in GraphPad and thematic extraction of free-text responses by three independent coders. |
| **Drake, 2023** | Academic medicine (academic medical centre) | “to provide leadership and career development opportunities to faculty members identified as having emerging leadership potential” | Not reported | Elements of the programme draw from both Kouzes and Posner’s five practices of exemplary leadership, as well as a multi-level organisational framework, and span multiple leadership theories such as transformational leadership | Summative | Not reported | Pre; Post-imm: ACCLAIM participant survey (Likert + open comments) administered at the start and end of the 9-month programme; institutional HR promotion data drawn separately across nine cohorts. | Participant surveys (Likert items plus open comments on leadership skills, confidence and application); institutional HR promotion data drawn separately for the nine-cohort programme window. |
| **Fassiotto, 2024** | Academic medicine (Stanford Medicine, eight departments) | “developed to reduce isolation among physician leaders from URGs in academic medicine leadership by building a supportive network of peers” | Social support and social network theory; psychological safety (Edmondson) | Programme logic model specified | Formative and summative | Logic model | Post-long (annual surveys, Years 1-5): self-developed surveys administered annually for five years to a single cohort (N=10; Year-1 n=8, Year-5 n=9) alongside institutional scholarly-productivity, promotion and recognition data. | Self-developed SMART surveys (Likert questions specifically designed around SNAP's programme goals; plus open-text prompts); qualitative content analysis of free-text responses; self-reported scholarly productivity, promotion and recognition data collected annually. |
| **Ford, 2021** | Academic medicine and academic STEM (national fellowships, Drexel University) | “increase the number of women leaders in academic health centres and to sustain their success”. | Not reported | Both programmes attend to published models of career development for women faculty leaders and to institutional practices that promote change and inclusion. | Summative | Leadership Learning Community Development framework | Pre; Post-short (6-8 wk); Post-long (2-year follow-up): Leadership Learning and Career Development (LLCD) survey administered online via Qualtrics at three waves; baseline within 4 weeks before the first on-site session, 6-8 weeks after graduation, and 2 years post-completion. | Revised LLCD survey* (24 items on importance and self-confidence across four leadership competencies; Strategic Finance, Organisational Dynamics, Communities of Leadership Practice, Personal and Professional Leadership; using a 1-7 numeric scale, plus eight items on professional-development activities and leadership advancement using yes/no and open-ended responses); Cronbach's alphas 0.76-0.90; two-way mixed-effects ANOVAs across programme (ELAM vs ELATES) and time; institutional promotion data drawn separately. |
| **Grando, 2022** | Academic medicine (biomedical informatics professional association) | “help participants develop leader identity and apply their leader identity to situations of social influence, interpersonal relationships, and team dynamics in an organisational context” | The programme’s curricular framework had 3 components: (1) Leading Self and Leading Ethically,18-20 (2) Power and Leadership and Social Capital, and (3) Leading Others in Change and Leadership Skills. | Not reported | Summative | Not reported | Pre; Post-session; Post-imm: pre-programme self-report survey administered before the year-long Women in AMIA Leadership Program and repeated at programme completion; post-session surveys administered after each onsite and virtual session; a post-programme impact survey was also administered at programme completion. | Pre- and post-programme self-report surveys assessing preparedness for leadership across 10 learning objectives, including leadership strengths, career vision, leadership confidence/presence, ethical decision-making, navigating structural barriers, influencing decisions, group decision-making, preparing teams for change, conflict management and use of financial reports; items were rated on 5-point Likert scales and pre-post responses were compared using paired t tests. Post-session surveys assessed session content, structure, delivery, materials and facilitation; a post-programme impact survey assessed leadership vision, career impact, upward mobility and coaching experiences. |
| **Heilemann, 2024** | Policing (Australian police organisation) | to strengthen career capital through three interdependent ways of knowing: knowing-why, knowing-how and knowing-whom. | Not reported | Career Capital Framework | Summative | Not reported | Post-short (1 mo; 4 mo; 9 mo); semi-structured participant interviews at 1 month post-programme; anonymous online VICPOL survey at 4 months; focus group and additional open-ended survey at 9 months. | Semi-structured participant interviews examining career capital, leadership behaviour, sponsorship and workplace application; anonymous programme-satisfaction survey; focus group and open-ended follow-up survey. VIA Signature Strengths Survey was used as a programme development activity rather than as an evaluation outcome measure. |
| **Helitzer, 2016** | Academic medicine (national career development programmes) | EWIM and MWIM “directed at building skills and confidence critical to advancement at each of the specific career stages” and ELAM increase the numbers and effectiveness of senior women leaders in health science organisations.” | Not reported | Not reported | Summative | Not reported | Post-long (retrospective alumnae assessment; intervals extending ≥1 year): 45 alumnae interviewed by telephone in 2011 reflecting on programme experiences from 1988-2010, sampled in 5-year cohorts; interval since programme therefore varies from ≤1 year to ≥20 years. | Web-based retrospective survey followed by semi-structured telephone interviews with former EWIM, MWIM and ELAM participants; interviews explored programme influence on skill development, networking, career advancement and institutional support. Myers–Briggs and 360-degree assessments were described as programme self-awareness activities rather than research outcome measures. |
| **Herbst, 2024** | Academic higher education (South African university) | “aimed to prepare emerging women leaders and middle managers for middle and senior leadership positions.” | Not reported | Not reported | Summative | Not reported | Pre; Mid; Post-immAdapted PwC (2014) Communicating with Confidence survey administered before the programme commenced and again after completion of the final development module; qualitative reflections/open-ended responses were collected during the year-long programme and at programme completion. | Adapted PwC (2014) Communicating with Confidence survey* assessing Point of View, Presence and Preparation, with a 7-point overall programme-impact item; Cronbach’s α = 0.903. Qualitative data were collected through participant reflections/open-ended responses and analysed using qualitative content analysis aligned with transformative learning. |
| **Hopkins, 2022** | Nonprofit / human services (university-delivered certificate programme) | “Developed in response to the continued growth of the health and human service and nonprofit sectors and critical need for diverse and competent leaders” | Not reported | Transformational leadership theory. | Summative | Not reported | Retrospective/post-only: Qualtrics online survey issued to programme entrants and again after certificate completion; coach interviews and coaching-circle reflections gathered in parallel across the first five programme years. | Mixed online Qualtrics surveys combining closed items with open-text prompts about individual, collective and organisational impact; coaching-circle reflections and facilitator notes; interviews with two peer-coaching facilitators (women of colour) for triangulation. |
| **Jones, 2025** | Academic medicine (multi-institutional, US; VA/university partnership) | “to provide leadership training and peer mentoring in a virtual, scalable format… widely accessible to women leaders in academic medicine” | Not reported | Not reported | Formative and summative | Not reported | Mid (programme midpoint); Post-imm (after completion): semi-structured participant interviews conducted mid-programme and at completion of the ≈12-month EMPOWER peer-circle programme; faculty-advisor focus groups run at completion. | Semi-structured individual interviews with programme participants (n=34 of 89) and focus groups with 14 faculty advisors (ELAM alumnae); Framework Method analysis to preserve within-case and cross-case comparison. |
| **Jowett, 2024** | Sport (coaching; UK high-performance pathway) | aimed to increase the number of women coaches, provide continuous professional development, raise awareness about gender bias, emphasise the importance of inclusivity, and ensure representation at all levels and types of sport | Not reported | Lens of stereotypical bias and specifically second-generation bias | Summative | Kirkpatrick | Post-imm; Post-long (3-year follow-up): online survey and semi-structured interviews at programme completion; further interviews at 3 years post-programme with a retained sub-sample. | Bespoke online survey and semi-structured participant interviews addressing coaching capability, leadership identity and career transitions in high-performance coaching contexts. |
| **Kang, 2024** | Academic medicine (US academic health system) | “was designed for professional development focusing on leadership, staff engagement, and joy and well-being at work” | Not reported | Transformational leadership | Summative | Kirkpatrick (implicit) | Pre + Post-session + Post-short (1 mo, 3 mo follow-up): baseline REDCap survey (leadership self-efficacy, motivation to lead, well-being) at introductory session; per-session satisfaction surveys; follow-up outcome surveys at 1 month and 3 months after the final session. | Closed electronic surveys via REDCap combining the Leadership Self-Efficacy Scale* (Kane & Baltes, eight items, 5-point Likert; adapted from Chen et al., 2001 in some reports), the Motivation to Lead Scale* (Chan & Drasgow, 2001; five items on affective-identity motivation, 5-point Likert) and the Mayo Clinic Well-Being Index* (Diener et al.-informed items); descriptive analysis of paired responses. |
| **Kelly, 2021** | Academic healthcare (US academic health institutions) | “contribute to the creation of healthier, more equitable work experiences for women physicians. WEL sought to make change at both individual and organisational levels, by fostering the development of women leaders and building an enduring infrastructure” | Not reported | Not reported | Formative and summative | Kirkpatrick (implicit) | Post-imm (per series + end): post-programme surveys administered at the end of each of the three WEL series (wellness, equity, leadership) and at overall programme completion; in-depth telephone interviews at end. | Post-programme surveys after each of the three WEL series plus a demographic survey (n=13); in-depth telephone interviews at overall programme completion; thematic analysis of interview data. |
| **Knipfer, 2017** | Academic STEMM (three German research organisations) | It aims to enhance women’s motivation to lead, increase their knowledge about academic leadership, and empower them to seek the support they need to proactively work towards appointment to a professorship, avoiding the “fix-the-women” approach. | Not reported | Campbell’s (1990) framework | Summative | Bespoke evaluation framework | Pre/per-module; Post-imm; Post-short (2 mo): module start/end questionnaires (including the Multifactor Leadership Questionnaire delivered as an online survey) for each of the four modules of the 8-month programme; follow-up assessment 2 months after the final module. | Module start/end questionnaires (bespoke items on knowledge, self-efficacy, motivation to lead, leader identity, and transfer intentions) plus the Multifactor Leadership Questionnaire* (MLQ; Bass & Avolio) administered as an online 360-style survey with participants and their team members providing ratings. |
| **Kvach, 2017** | Academic medicine (Ethiopian medical schools; US university partnership) | “to promote gender equity as a condition of the grant award.” | Not reported | Not reported | Summative | Bespoke evaluation model | Post-imm: focus groups and semi-structured interviews conducted after fellowship completion (fellowship comprised a 2-week intensive plus annual in-person follow-up visits in Ethiopia for ≥1 year). | Focus groups and semi-structured interviews with fellows and male institutional leaders in Ethiopia; thematic analysis of qualitative data emphasising gender equity, mentoring and institutional context. |
| **Lämsä, 2019** | Professional / private sector (Finnish executive education) | “to increase gender equality in leadership and entrepreneurship, support women in career development and finding meaningful work, strengthen women’s identities as leaders, and support women’s professional networking” | Not reported | Theory of self-efficacy, Sen’s (1985) capabilities approach, and human capital theory | Summative | Not reported | Pre; Post-long (18 mo): face-to-face interviews at the beginning of the 2.5-year fMBA programme and again approximately 18 months after programme completion. | Open-ended/semi-structured participant interviews examining programme goals, leadership roles, career strategies, career barriers, gender, learning, leadership competency development, career advancement and networking; interviews were recorded, transcribed verbatim and analysed using qualitative content analysis, with some simple quantification of outcomes. |
| **Lee, 2024** | Academic medicine (Johns Hopkins University School of Medicine) | “to support the leadership development of early career women faculty” | Not reported | Not reported | Formative and summative | Not reported | Pre + Post-imm (+ 1/6/12-month participant-set action plans): pre-programme Qualtrics questionnaire (Likert + open-ended) issued in the month before ECWLP; post-programme questionnaire on the final day of the programme with follow-up emails over 6 weeks; 1-, 6- and 12-month action plans generated by participants at the end of the programme rather than collected at those subsequent timepoints. | Pre- and post-programme questionnaires with Likert-type items across five categories (self-rated skills and confidence in leading; confidence in applying basic leadership coaching principles; conflict or alignment between pursuing leadership and competing factors; barriers to career advancement; desired leadership positions) plus open-ended items; Wilcoxon signed-rank tests on matched pre-post responses with Bonferroni correction; content analysis of open-ended responses; participant-generated 1-, 6- and 12-month action plans elicited at programme end. |
| **Martínez-Martínez, 2021** | Corporate, multiple sectors (Spain; national programme) | “identify and promote women’s talents by developing and strengthening their professional and leadership skills and abilities, as well as facilitating their access to senior management positions in companies” | Not reported | Authentic leadership theory | Summative | Not reported | Post-long (retrospective, 1–6 years): semi-structured interviews conducted after participants had been promoted (≥1 year post-programme completion, as reported); face-to-face, phone or online. | Semi-structured participant interviews (face-to-face, phone or online) with alumnae who had been promoted; thematic analysis with an authentic-leadership lens. |
| **Mbilishaka, 2023** | Academic higher education (psychology faculty) | “This programme aimed to close the gendered leadership by (a) creating a more intentional opportunity for building sustained community, peer and mentor supports, advocacy, research, and practice partnerships; (b) disrupting the persisting disparities faced by women; and (c) providing more explicit support for participant narrative skill development” | Not reported | Not reported | Summative | Not reported | Retrospective (post-programme autoethnography): three Black women faculty participants engaged in reflective self-study post-programme; weekly self-reflection diaries and group reflection sessions were data sources during the 10-week DiLH programme. | Weekly self-reflection diaries during the 10-week programme; group reflection sessions after each weekly meeting; autoethnographic co-analysis of diaries and session transcripts. |
| **Megheirkouni, 2017** | Sport management (UK sports organisations) | Seeks to achieve level of key performance indicators identified by the management team to: develop leadership behaviour, capabilities, and confidence of all participants; provide development opportunities through the adopted programme; support change in all participants; and support and build strong networking among participants | Not reported | Transformational learning theory | Summative | Not reported | Retrospective (post-only): semi-structured telephone interviews with 10 of 15 programme participants after completion of the 3-year pilot; interval since programme not specified. | Semi-structured telephone interviews with 10 of 15 programme participants (women in middle and senior management across UK sports organisations); thematic interview analysis. |
| **Miles-Cohen, 2020** | Healthcare (American Psychological Association, professional association) | “to prepare, support and empower women psychologists, including those of diverse backgrounds, as leaders to promote positive changes in institutional, organizational, and practice settings as well as APA and SPTA governance.” | Not reported | Not reported | Summative | Not reported | Mid programme; Post-imm; Post-long (retrospective): qualitative and quantitative evaluations completed throughout the 5 days of training and for the overall LIWP experience; Post-Institute Self-Evaluation of Goals completed after training; formal alumnae survey administered from October 2017 to capture leadership accomplishments following programme completion, including an annual survey covering October 2017-September 2018. | Qualitative and quantitative module/programme evaluations; ratings of leadership-development interests; Post-Institute Self-Evaluation of Goals assessing strengths, challenges, needs, goals and accomplishments; alumnae survey assessing awards, leadership/service roles, promotions/employment changes and perceived LIWP contribution. Leadership Skills Self-Efficacy Scale* and Fundamental Interpersonal Relations Orientation–Behavior. * were used as programme developmental/self-awareness assessments rather than clearly reported pre-post evaluation outcomes. |
| **Mohanakumar, 2022** | Public administration / customs (Asia-Pacific, multinational) | “build their leadership skills [specifically an adaptive mindset], bridge the gender gap and prepare themselves to be future customs leaders” | Not reported | Adaptive leadership theory. | Summative | Not reported | Post-imm: online survey (Likert + open-ended) plus reflective essays completed at the end of the 4-week CCP-WPDP training. | Online survey (Likert items on leadership capability, adaptive mindset and gender equity plus open-ended items); participant reflection essays authored by course completers. |
| **Nakamura, 2022** | Professional, multiple sectors (chemicals, pharmaceuticals, higher education, manufacturing, media, R&D) | The programme was designed to develop women in leadership | Not reported | Authentic leadership framework | Summative | Not reported | Mid; Post-imm; archival pre-programme material: pre-programme survey during the 3-day intensive; individual interviews conducted within 1 month of programme completion; field observations and archival programme materials analysed alongside. | Individual case-study interviews (leadership stories, programme experience, authenticity); field observation during the 3-day intensive; facilitator reflections; document review of programme materials including pre-programme survey and agenda. |
| **Nash, 2018** | Academic STEMM (international programme, Australia-run) | “The leadership programme seeks to address the underrepresentation of women in science leadership and in climate change decision-making” | Not reported | Not reported | Summative | Not reported | Mid; Post-short (<2 mo): participant video diaries recorded during the 3-week Antarctic voyage (mid-programme) and immediately after return. | Participant video diaries capturing lived experience during and immediately after the Antarctic voyage; qualitative analysis of video content and voiceover reflection. |
| **Nash, 2021** | Academic STEMM (international programme) | “enhancing and developing the leadership, strategic and scientific capabilities of women in STEMM fields… and address the underrepresentation of women in science leadership” | Not reported | Transformational learning theory. | Summative | Theory of change | Mid; Post-long (1-year follow-up): video diaries recorded during the Antarctic voyage; semi-structured interviews with the same cohort at ≈1 year post-voyage (longitudinal identity study of the 2018 cohort). | Video diaries (voyage and immediate post-voyage) combined with semi-structured interviews at 1-year follow-up; longitudinal identity analysis tracing shifts in leader self-concept. |
| **Nowling, 2018** | Academic medicine (US academic medical centre) | “improving the number of women in the upper ranks and in leadership positions.” | Not reported | Not reported | Summative | Kirkpatrick | Pre; Post-imm; Post-long (1–4 years): pre-programme surveys assessed topics of interest, knowledge of career track and leadership interest; post-programme surveys assessed reaction, learning and intended application of knowledge/skills. A follow-up survey in 2017 assessed promotion outcomes among 2013–2016 attendees, and institutional HR data compared promotion rates during the 4-year pre-CDP period (2009–2012) with the 4-year post-CDP period (2013–2016). | Pre- and post-programme attendee surveys assessing leadership interest, awareness of career track, satisfaction, perceived knowledge/skills gained, intended application of learning and promotion planning; 2017 follow-up survey assessing applications for and attainment of promotion; institutional HR promotion data for approximately 3000 faculty comparing pre-CDP (2009–2012) and post-CDP (2013–2016) promotion rates by gender and academic rank. |
| **O’Brien, 2022** | Outdoor education (Outward Bound, UK) | ‘to create a nurturing environment with opportunities for personal progression towards the technical, intrapersonal and interpersonal skills required to work within Outward Bound and progress within the outdoor sector’ | Not reported | Transformational learning theory | Summative | Not reported | Post-imm: semi-structured participant interviews conducted after completion of the 10-week Outward Bound residential course. | Semi-structured participant interviews after Outward Bound course completion; grounded-theory analysis of transformative learning through outdoor leadership. |
| **Parker, 2018** | Academic higher education (Australian university) | “support women’s advancement from senior lecturer to associate professor” | Not reported | Not reported | Formative and summative | Not reported | Retrospective cross-sectional/post-only (programme cohorts 2010–2016): single online SurveyMonkey survey administered across programme cohorts (participants completed the programme 2010-2016); pilot with 17 respondents plus 6 follow-up face-to-face interviews informed survey design. | Online SurveyMonkey survey (11 features of the programme rated on a 5-point Likert scale from "not beneficial" to "extremely beneficial" plus career-application questions and open-text) with 6 follow-up face-to-face interviews within a pilot of 17 respondents used to refine the survey. |
| **Pelfrey, 2022** | Academic medicine (Cleveland Clinic academic medical centre) | “explicit goal of increasing the number of women in visible leadership positions in academic medicine and health sciences” | Not reported | Social Cognitive Theory and Social Network Theory | Summative | Not reported | Pre; Mid; Post-imm; Post-long (retrospective, 1–8 years): pre- and post-programme surveys and programme application data were collected as part of the wider FLEX evaluation; open-ended survey data captured experiences during training and at programme completion; semi-structured interviews were subsequently conducted with 25 graduates from seven cohorts, 1–8 years after FLEX participation. | Mixed qualitative evaluation data comprising programme application forms, fixed- and open-response pre- and post-programme surveys, programme data and semi-structured interviews with 25 FLEX graduates. Interviews used eight core questions with probes addressing programme experience, behavioural change, leadership vision, networking, executive coaching and ongoing support, and were analysed using two-stage open and axial coding informed by a constructivist grounded-theory approach. |
| **Peterson, 2019** | Academic higher education (Swedish universities; national IDAS programme) | “set up to support women and increase the proportion of women senior academic leaders.” | Not reported | Not reported | Summative | Not reported | Post-long (retrospective alumni assessment): semi-structured interviews with 15 IDAS alumnae who had become university rectors; retrospective reflection on programme influence, interval since programme varies. | Semi-structured interviews with 15 IDAS alumnae who had become university rectors; hermeneutic analysis of career-authority narratives. |
| **Round, 2024** | Professional, multiple sectors (Primarily financial services and manufacturing) | “Aimed to develop participants’ skills, connect individuals within a supportive network, and provide positive role models” | Not reported | Not reported | Summative | Not reported | Pre; Post-imm: closed pre-programme survey (Psychological Capital scale, demographics, leadership efficacy, motivation, identity) and post-programme survey at end of the 6-month online JAEFLP. | Closed pre- and post-programme surveys including the Psychological Capital (PsyCap) scale*(Luthans et al.) with items on hope, self-efficacy, resilience and optimism, plus demographic, motivation to lead and leader-identity items. |
| **Russell, 2023** | Academic and professional association (family and consumer sciences, US) | “Developed to create a pool of competent future leaders to drive and support the organisation” | Not reported | Transformational leadership and servant leadership | Summative | Not reported | Retrospective (post-only across mixed cohorts): focus groups and interviews with 25 informants (programme participants, mentors and trainers) drawn across multiple cohorts of the AAFCS Leadership Academy; timing since programme not specified. | Focus groups with programme participants and separate interviews with mentors, trainers and executive-team members; mixed thematic analysis of the two data streams. |
| **Ryan, 2021** | Healthcare (Irish hospital) | “focused on developing healthcare leaders through the lens of leader identity” | Not reported | Not reported | Summative | Not reported | Mid; Post-imm: interviews, focus groups, critical-incident diaries, exit surveys and observations collected across the 18-month programme; exit survey and final interviews at completion, critical-incident diaries and observations throughout. | Interviews (25 women; 37+ hours of recordings), focus groups (7 workshops; 14+ hours), critical-incident diaries (194 written online incidents with guided reflective prompts), individual exit surveys (25 open-ended responses to three questions) and observational notes from full-day seminars and peer-group discussions; NVivo 12; constant-comparative method with theoretical sampling. |
| **Schwartz, 2021** | Academic medicine (Society for Pediatric Anesthesia, national society) | “multidimensional programme to help address the significant career development, leadership, and promotion gender gap between men and women in anesthesiology” | Not reported | Not reported | Summative | Not reported | Retrospective cross-sectional (ongoing programme; survey at ~28 months after programme launch): anonymous survey of WELI members with variable programme exposure due to rolling admission; no fixed ≥12-month post-completion or post-baseline follow-up timepoint. | Anonymous online Qualtrics survey with 5-point Likert items assessing WELI component impact (dyads, workshops, online content, networking) across career-development areas; project completion, goal-setting, mentorship abilities, career advancement, well-being plus three free-text prompts; descriptive analysis in GraphPad; qualitative thematic analysis by three coders with reflexivity checks. |
| **Sears, 2019** | Healthcare (multispecialty health system, Texas) | as a tool to fight burnout among female physicians | Not reported | Not reported | Formative and summative | Not reported | Pre-development; Post-imm; Post-short (6 mo): a programme-development survey was administered before WLiM launched to inform programme design; post-leadership-summit evaluation surveys were administered following programme events; a second programme evaluation survey was administered approximately 6 months after the initial development survey/programme launch. | Bespoke programme-development and evaluation surveys containing quantitative and open-ended items. The pre-launch survey assessed interest in short- and long-term leadership development, interest in WLiM, private social-media participation, organisational support for leadership development and barriers to leadership. Post-programme surveys assessed programme content and usefulness, networking, leadership skills and future programme needs; quantitative data were summarised descriptively and responses to eight open-ended questions were coded inductively into thematic categories. |
| **Sears, 2025** | Healthcare (Baylor Scott & White Health system) | Programme aim not reported; the evaluation aimed ‘to determine if a shared-learning, social-based leadership development programme will affect burnout and career trajectory for female physicians’.” | Not reported | Not reported | Formative and summative | Not reported | Pre + Mid + Post-imm: multi-dimensional questionnaires distributed via REDCap at three timepoints across two years (03/2018-04/2018 and 01/2020), covering Maslach Burnout Inventory subscales, Areas of Worklife Survey and leadership-aspiration items. | Interest-in-leadership survey, intent-to-retain-current-employment survey and open-text comments; single- and three-item abbreviated Maslach Burnout Inventory-Human Services Scale for Medical Personnel (MBI-HSS-MP)* subscales for emotional exhaustion, depersonalisation and personal accomplishment; Areas of Worklife Survey (AWS)*; leadership-aspiration single Likert item (0-10). |
| **Selzer, 2017** | Academic higher education (urban, public, top-tier US university) | “Prepare mid-career faculty and staff women for senior leadership in higher education, and to identify participants’ readiness for higher-level positions” | Not reported | Not reported | Summative | Not reported | Post-imm (reflective autoethnography): three co-authors, all WLP participants, engaged in collaborative autoethnography after programme completion (reflective journals and group narrative essays generated during the writing process, not during the programme itself). | Collaborative autoethnography (CAE) among three co-authors: reflective journals, group narrative essays and iterated writing sessions; connected-knowing analytic frame with transformative-learning theoretical grounding. |
| **Sims, 2022** | Academic higher education (US university) | “implemented to help achieve institutional change and improve gender equity in a large U.S. university.” | Human Performance Technology (HPT) framework | Logic model and Kirkpatrick’s four levels of evaluation. | Formative and summative | Human Performance Technology; Logic model; Kirkpatrick | Pre; Post-imm: baseline surveys (MindGarden MLQ 360, Qualtrics for other items) at T1 (Fall 2017); T2 surveys (Qualtrics) in Spring 2018 at programme completion; semi-structured individual interviews in Spring 2018 post-programme. | Organisational reports (institutional documents including NSF ADVANCE materials); focus groups; semi-structured individual interviews (in-person or telephone with a $20 gift card); web-based surveys including the MLQ* (MindGarden, 45 items, 5-point Likert from “0"not at all"to 4"frequently") and the Gender and Leader Identity Interference scale* (Karelaia & Guillén, 10 items, 7-point Likert); per-session learning-satisfaction surveys (7-point Likert on goal attainment and applicability); Kirkpatrick four-level framework guiding synthesis. |
| **Skarupski, 2019** | Academic medicine (Johns Hopkins University School of Medicine) | “to promote experiential learning by incorporating interactive case studies, facilitated discussion, skills building using role-play, and reflective practice” | Not reported | Not reported | Summative | Not reported | Post-long (retrospective survey, 1–8 years; retrospective pre-post ratings): single retrospective online survey (May 2017) of 285 graduates across eight cohorts; retrospective pre-post self-assessment format with Likert items and open comments; interval since programme 1-8 years across the cohort. | Single retrospective online survey (May 2017, with reminders) using Likert items and open comments; retrospective pre-post self-assessment format asking respondents to rate perceived state before and after programme in one sitting; descriptive and inferential analysis across eight cohorts (285 graduates; 40% response rate). |
| **Spalluto, 2017** | Academic medicine (radiology department, US academic medical centre) | “(1) cultivate knowledgeable, successful, confident women prepared to achieve career success and assume leadership positions and (2) promote a departmental culture supportive of career advancement of women faculty members.” | Kellogg logic model | Not reported | Formative | Kirkpatrick | Pre; Mid. At the end of Year 1 an initial needs-assessment survey was conducted before programme implementation; module-specific pre- and post-surveys were administered around individual educational modules throughout the 2-year LIFT-OFF programme; a formative programme evaluation was conducted at the end of Year 1 to assess interim outcomes and inform Year-2 programme development. | Initial needs-assessment survey used to identify priority faculty-development topics; module-specific pre- and post-surveys assessing participant knowledge of the targeted subject matter across 14 educational modules; and an end-of-Year-1 formative programme evaluation assessing access to faculty-development and career-advancement opportunities, professional advancement and programme impact. Pre-post module responses were analysed for changes in knowledge, including statistical testing of changes in categorical responses. |
| **Sutton, 2024** | STEMM (Government research, National Cancer Institute, US) | “aims to empower women to explore careers across a broad range of fields, including academia, industry, and government, and to excel in leadership positions in those fields.” | Not reported | Not reported | Formative and summative | Not reported | Pre; Post-imm: Self-developed online surveys administered within 1 week of programme acceptance and within 1 week of programme completion; programme data were also examined across cohorts. | Self-developed pre- and post-programme survey assessing programme expectations, self-perceived skills, confidence in career pathways, interpersonal relationships, and programme satisfaction, using quantitative rating-scale and open-ended items. |
| **Valerio, 2024** | Academic STEM (Agricultural research) | “aims to support women agricultural researchers across the Indo-Pacific region improve their leadership and management skills.” | Transformative learning theory | Not reported | Formative and summative | Not reported | Post-imm: online survey and qualitative interviews with 15 Meryl Williams Fellowship participants after programme completion; secondary document analysis of Fellowship reports supplied contextual data. | Online survey (open-ended and closed items on programme experience and career capital); semi-structured qualitative interviews with 15 Meryl Williams Fellowship alumnae analysed thematically in NVivo 12; secondary document analysis of Fellowship reports for triangulation. |
| **Van Oosten, 2017** | Academic STEM and STEM Corporate (executive education delivered via university partnership) | “aimed at increasing the advancement and retention of women in the STEM professions” | Not reported | Intentional Change Theory (Boyatzis, 2006) | Formative and summative | Kirkpatrick | Post-imm: Post-programme participant feedback and interviews across five cohorts (three STEM, two manufacturing); 360-degree emotional-intelligence assessment used as a programme development component rather than as an evaluation outcome. | Post-programme surveys and interviews capturing job promotions, awareness of unconscious bias, personal transformation/self-efficacy, and coaching benefits; 360-degree emotional-intelligence assessment used as a programme learning component rather than as an independent research measure. |
| **Walsh, 2022** | Academic Medicine and global health (US institution with LMIC partners) | “to promote the advancement of female global health research professionals and prepare them for leadership positions” | Not reported | Logic model | Summative | Logic model | Pre; Post-imm: quantitative surveys administered at start and end of the year-long pilot; supervisory-endorsement data captured at enrolment; evaluative feedback form at completion | Quantitative surveys (Likert items on research skills, mentoring adequacy and global-health leadership); evaluative feedback form completed at programme end; supervisory-endorsement data captured at enrolment as an institutional-support indicator. |

***Note*.** Programme aims and content are reported using the terminology of the original authors and have been lightly edited for clarity and consistency. “Not reported” indicates that the study did not explicitly identify the relevant model, theory of change, or evaluation framework. Data-collection timepoints are coded as pre-programme (Pre), during programme (Mid), immediate post-programme (Post-imm), short-term follow-up within 12 months (Post-short), long-term follow-up at 12 months or longer (Post-long), or retrospective post-only where participants reflected after programme completion without a prospective baseline. Formative evaluation refers to data used to inform programme development or refinement; summative evaluation refers to assessment of programme outcomes or impact. An asterisk (*) denotes a named validated or established psychometric instrument. AAMC, Association of American Medical Colleges; CRAI, Clinical Research Appraisal Inventory; ELAM, Executive Leadership in Academic Medicine; EWIM, Early-Career Women Faculty Professional Development Seminar; HR, human resources; LLCD, Leadership Learning and Career Development; LMIC, low- and middle-income country; MLQ, Multifactor Leadership Questionnaire; MWIM, Mid-Career Women Faculty Professional Development Seminar; PsyCap, Psychological Capital; REDCap, Research Electronic Data Capture; STEM, science, technology, engineering and mathematics; STEMM, science, technology, engineering, mathematics and medicine; WELI, Women’s Empowerment and Leadership Initiative.

# Supplementary Table S4. TIDieR mapping of extracted programme characteristics

| **TIDieR item** | **Extraction field** | **Location** | **Reporting across included studies** |
| --- | --- | --- | --- |
| 1. Brief name | Programme name | Table 2 | A programme name or identifying label was reported in 52/57 study reports. This included both formally branded programme names and generic programme titles |
| 2. Why (rationale, theory) | Programme aim/content; theoretical/pedagogical model; theory of change | Supplementary Table S3 | A programme aim or rationale was reported in 57/57 study reports. An explicit theoretical or pedagogical model informing programme design was reported in 7/57 reports. An explicit or inferable programme logic or theory of change was identified in 23/57 reports, although formal author-labelled theories of change were uncommon. |
| 3. What (materials) | Programme materials or resources, such as manuals, curricula, workbooks, assessments or other resources, where described | Supplementary Table S3, where reported | Programme materials and resources were not systematically extracted and were inconsistently described. |
| 4. What (procedures) | Programme activities and components, including workshops, seminars, coaching, mentoring, networking, peer learning and reflective activities | Not systematically extracted | At least one programme procedure or activity was described in 57/57 study reports, although the completeness and level of detail varied substantially. |
| 5. Who provided | Programme provider, facilitator, coach, mentor or trainer details, where reported; Cost/Funding | Supplementary Table S8 | Programme cost or funding information was reported in 41/57 study reports. Provider and facilitator characteristics were not systematically extracted and were inconsistently reported. |
| 6. How (mode of delivery) | Delivery mode, including in-person, online, virtual, hybrid, residential or distance-based delivery | Supplementary Table S8 | Delivery mode was explicitly reported in 24/57 study reports. In 32/57 the likely delivery mode could be inferred from the described activities but was not clearly reported. In 1/57 it was not clear the delivery mode. |
| 7. Where | Country; Sector | Table 2 | Country and sector were identifiable in 57/57 study reports. Specific delivery settings or venues were less consistently reported. |
| 8. When and how much | Overall programme duration, frequency, intensity and intervention dose | Table 2 | Overall programme duration was reported in 55/57 study reports and was unclear for Martínez-Martínez et al. (2021) and Russell et al. (2023). Session frequency, contact hours and total intervention dose were less consistently reported. |
| 9. Tailoring | Personalisation or adaptation of programme content or delivery to participants’ needs, roles, career stages or contexts, where reported | Where reported in Supplementary Table S8 | Some programmes target specific career stages or use individual goals, coaching plans, assessments or projects, but adaptations to each participant are rarely described systematically. |
| 10. Modifications | Changes made to the programme during delivery or across cohorts | Where reported in Supplementary Table S8 | Programme modifications were not systematically extracted and were rarely reported. |
| 11. How well: planned | Planned strategies for maintaining intervention fidelity, adherence or delivery quality | Not systematically extracted | Planned fidelity, adherence and delivery-quality strategies were not systematically extracted and appeared to be rarely reported. |
| 12 How well: actual | Fidelity, adherence, attendance or programme delivery achieved in practice | Not systematically extracted | Attendance or programme completion was occasionally reported. Fidelity to planned programme content or delivery was not systematically extracted and appeared to be rarely assessed. |

***Note*.** TIDieR items 3, 5 and 9-12 were not represented by dedicated extraction fields and were inconsistently or rarely reported in the source studies. This incomplete intervention reporting limits programme replication and interpretation and is addressed in the Discussion. TIDieR = Template for Intervention Description and Replication

# Supplementary Table S5. Equity-relevant participant characteristics and programme accessibility mapped to PROGRESS-Plus

| **Study** | **Place of residence; delivery mode** | **Race/ethnicity/culture/language** | **Occupation, including career stage** | **Gender/sex** | **Education** | **Socioeconomic status, including proxy indicators** | **Social capital, including proxy indicators** | **Plus: personal characteristics associated with discrimination** | **Plus: relationship and family characteristics** | **Programme accessibility and participation support** | **Programme funding** | **Integration within a broader equity initiative** |
| --- | --- | --- | --- | --- | --- | --- | --- | --- | --- | --- | --- | --- |
| **Adeola et al. (2024)** | USA; virtual platform | White 60%; Asian 20%; Black/African American 20% | Nurse scholars; 80% PhD-prepared; 60% on tenure-track faculty appointments | Women | PhD-prepared 80%; 80% US-educated | NR | MNRS executive leaders selected five experienced and diverse nurse scholars (not open application) | NR | NR | MNRS executive leaders selected five experienced and diverse nurse scholars (not open application); NR. | NR | No |
| **Barnard et al. (2022)** | United Kingdom; in person | White (British/Irish/other) majority; Black and Minority Ethnic (BME) 5.7% | Early- and mid-career participants in UK higher education; academics 53.0%, professional services 47.0% | Women | NR | NR | Places funded or sponsored by employing institutions | Reports LGBT 6.9%; disability/hearing impairment 8.8%; registered disability 1.2% | NR | Open enrolment to any woman in higher education; places funded or sponsored by employing institutions. | Advance HE programme; institutionally purchased places | Yes |
| **Brixey et al. (2025)** | USA; in person | NR | Academic health faculty and trainees; 54.3% in training | Men and women attended | Radiology/medical physics faculty (MD, PhD/DABR) and trainees (residents/fellows). | NR | NR | Age 30-39 54.3% | NR | NR. | Department-supported. | No |
| **Brue & Brue (2016)** | USA; in person | NR | Programme alumnae; job experience 10-22 years (average) | Women | NR | NR | NR | NR | NR | Study participants recruited by programme director email invitation; self-selected volunteers | NR | No |
| **Brue & Brue (2018)** | USA; in person | NR | Programme alumnae | Women | NR | NR | NR | NR | NR | NR. | NR | No |
| **Chang et al. (2016)** | USA; in person | NR | Faculty in US academic medicine; MD, PhD or MD/PhD; assistant to full professor | Women | Assistant professor participants were 82% MD, 12% PhD, and 6% MD/PhD; associate professor participants were 77% MD, 17% PhD, and 6% MD/PhD; and full professor participants were 59% MD, 35% PhD, and 7% MD/PhD. | NR | Competitive selection | Mean age at appointment 35.2–44.7 across ranks. | NR | Competitive selection; NR. | AAMC-sponsored national programmes | Yes |
| **Chang et al. (2020)** | USA; in person | Race/ethnicity not considered | Faculty; 64% assistant professors, 36% associate professors | Women | Assistant-professor participants: 82% MD, 12% PhD, 6% MD/PhD; associate-professor participants: 77% MD, 17% PhD, 6% MD/PhD | NR | Faculty nominated and selected from women recognised for academic success and leadership potential | NR | NR | Faculty nominated and selected from women recognised for academic success and leadership potential; NR. | AAMC-sponsored national programmes | Yes |
| **Chasserio & Bacha (2024)** | France; in-person | “mainly middle- and upper-class white, cisgender women” | Participants from 14 cohorts, mean age 42 (34-54); 36 in professional/managerial roles and 11 unemployed at programme entry (as reported) | Women; described as mainly cisgender women | Bachelor’s and master’s degrees (described as relatively high level of education) | 36 in professional/managerial roles and 11 unemployed at programme entry (as reported); 11 participants unemployed at programme entry (career returners); tiered pricing widened access (€3,500 for employed women funded by employers | Interview selection | Age 34-54 (mean 42) | Aimed to include women in transition after long career interruptions (for the most part, periods spent caring for children | Interview selection; tiered pricing widened access (€3,500 for employed women funded by employers; €550 for women in transition paying personally). | NR | No |
| **Chaudron et al. (2021)** | USA; in person | NR | Mid-career faculty; 49 associate professors; 69% held an MD or DO | Women | 69% MD or DO; 31% PhD, EdD, DNSc or MS. | no cost to the individual or department; total programme cost approximately $7,500 per cohort | Nominated and selected based on leadership potential | NR | NR | Nominated and selected based on leadership potential; no cost to the individual or department. | Institutionally funded; total programme cost approximately $7,500 per cohort | No |
| **Cordeiro et al. (2024)** | USA & LMICs; hybrid | NR; country-income location reported 72% LMICs, 27% US) | Global health early-career researchers; 72% from LMICs, 27% US; early/mid-career investigators | Women | Highest degree: Bachelor’s 9%, Master’s 50%, MD and/or PhD 41%; 90% carried an MD, PhD or other doctoral degree | Programme described as"low cost." | NR | Median age 40 (IQR 32-44) | currently married 91%; mean children 2.5 (SD 1) | NR | Programme described as"low cost." | Yes |
| **Danhauer et al. (2019)** | USA; in person | White (non-Hispanic) 73.9%; Asian/Pacific Islander 12.3%; Black 7.7%; Hispanic 3.1% | Early-career faculty; assistant professor 87.7%; MD/equivalent 63.0% | Women | MD or equivalent 63.0%; 16% other doctoral-level degree | department chairs provided release time and covered tuition costs | department chairs provided release time and covered tuition costs | Age 30-39 75.4% | married/partnered 86.2%; 2 children 32.3%; caring for aging parents 24.6% | Competitive application process; department chairs provided release time and covered tuition costs. | Institution and departments | Yes |
| **David et al. (2025)** | USA; online components | White 54%; Asian/Asian American 23%; Black/African American 12%; multiracial/other 12%; non-Hispanic 92%. Most participants were US citizens | Early-career faculty; assistant professor rank 69%; full-time faculty; at Yale approximately 5 years | Women | NR | full-time faculty; approximately US$230 per participant (US$3,700 annually) covered by the YSM Office of Faculty Development | Initially nominated then expanded to an open call for early-career women faculty | heterosexual majority; LGBTQIA+: No 24, Yes 1, prefer not to say 1; Most participants were aged 35-44 years | NR | Initially nominated then expanded to an open call for early-career women faculty; approximately US$230 per participant (US$3,700 annually) covered by the YSM Office of Faculty Development. | Yale School of Medicine Office for Women in Medicine and Science + Office of Diversity, Equity, and Inclusion | Yes |
| **DeFrank-Cole et al. (2017)** | USA; in person | NR | Faculty and staff (including Provost’s office staff working part-time on the programme) | Women | NR | Women faculty and staff (including Provost’s office staff working part-time on the programme); Programme described as"cost-effective."Funding source NSF ADVANCE IT | NR | NR | NR | NR | Programme described as"cost-effective."Funding source NSF ADVANCE IT | No |
| **Deutsch et al. (2022)** | USA; virtual components | NR | Paediatric anaesthesiologists: protégés (mostly assistant professors 60% and associate professors 36%), advisors and dual protégé-advisors | Protégés were all women, but advisors could be men or women | Medical degree required; MD or DO inferred from professional role. | Membership fees | Paediatric anaesthesiologists: protégés (mostly assistant 60% and associate professors 36%), advisors and dual protégé-advisors; Supervisor endorsement required; Protégés initially nominated by chief/chair, later expanded to self-nomination or nomination by other leaders (CV and personal statement); advisors nominated on experience and skills and matched by the advisory board; advisors and GAIN networking participated free of charge | NR | Attrition reasons cited at programme level included childcare and pregnancy | Supervisor endorsement required. Protégés initially nominated by chief/chair, later expanded to self-nomination or nomination by other leaders (CV and personal statement); advisors nominated on experience and skills and matched by the advisory board. Protégés paid US$450/year. | Society for Pediatric Anesthesia and Masimo support; membership fees | No |
| **Drake et al. (2023)** | USA; in person then changed to virtual delivery | NR, but aimed to recruit underrepresented Minorities | Faculty Scholars | Mixed-gender cohort; women 57% | NR | UNCSOM funded departments to cover scholars' protected time | Nominated participants selected by leadership as emerging leaders | NR | NR | Nominated participants selected by leadership as emerging leaders; UNCSOM funded departments to cover scholars' protected time. | Supported by the UNCSOM and the Office of Faculty Affairs and the Department of Medicine | Yes |
| **Fassiotto et al. (2024)** | USA; in person | Racially/ethnically diverse; 2-3 from each group (20.0-30.0%): Asian, Black/African American, Hispanic/Latinx, White | Physicians; at inception associate professors 70%, assistant professors 30%; clinician-educator line 50%, university medical line 50% | Women | NR | NR | Invited after identification by the Dean's Office as emerging leaders with prior leadership experience | NR | NR | Invited after identification by the Dean's Office as emerging leaders with prior leadership experience; NR. | Funded by the Stanford University School of Medicine Dean’s Office | Yes |
| **Ford et al. (2021)** | USA; in person | NR | Senior faculty fellows in academic medicine and engineering/STEM; year-long, part-time fellowships | Women | NR | Women-only, year-long, part-time fellowships | NR | NR | NR | NR. | Funded or sponsored places (institutional sponsorship). | Yes |
| **Grando et al. (2022)** | USA; in-person with remote instruction | White/Caucasian 70.8%; Asian 29.2%; Hispanic/Latina 8.3% | AMIA members; informatics professionals and academics; academic rank ranged from Research Fellow to Professor, with additional IT professional and clinical informaticist roles | Women | Doctorate 66.67%; Bachelor 12.50% (highest degree, as reported) | Tuition US$3,500 plus travel, largely covered through scholarships and organisational sponsors; Association-supported with sponsor-funded scholarships | Women AMIA members; Open application followed by selection; tuition US$3,500 plus travel, largely covered through scholarships and organisational sponsors | Age 25-64; most participants were aged 35-54 (70.8%) | NR | Open application followed by selection; tuition US$3,500 plus travel, largely covered through scholarships and organisational sponsors. | Association-supported with sponsor-funded scholarships. | No |
| **Heilemann & Parker (2024)** | Australia; NR | NR | Senior Sergeants and Inspectors with 6-34 years’ tenure; rural or urban posting recorded | Women | NR | NR | Nominated and selected for demonstrated leadership potential; allocated an internal VICPOL sponsor | NR | NR | Nominated and selected for demonstrated leadership potential; allocated an internal VICPOL sponsor. | NR | No |
| **Helitzer et al. (2016)** | USA & Canada; in person | NR | Faculty alumnae; 56% full professor, 27% associate professor; MD/PhD mix | Women | MD/PhD (degree mix reported) | Tuition and participation time covered | ELAM participants nominated by the Dean or other leaders, EWIM and MWIM recruitment NR | NR | NR | ELAM participants nominated by the Dean or other leaders, EWIM and MWIM recruitment NR; Tuition and participation time covered. | ELAM/EWIM/MWIM programmes themselves institutionally sponsored | Yes |
| **Herbst et al. (2024)** | South Africa; in person | None collected (authors preserved participant anonymity) | Academics and professional staff (emerging leaders); middle-level and aspiring leaders | Women | None collected | NR | NR | NR | Women's caregiving and work responsibilities were discussed qualitatively | NR. | University Capacity Development Grant (UCDG) from the Department of Higher Education and Training (DHET), South Africa | Yes |
| **Hopkins et al. (2022)** | USA; flexible; 76% were geographically situated in an urban area | Women of colour 56% (39% of these identified as black), 44% were white. | Professionals in human service nonprofit organisations; managers and emerging leaders; 61% were at manager level, 10% at CEO level, with 2-40 years of experience (average 19 years) | Women | 57% had a master’s degree or higher | Programme described as affordable | NR | Age range 31-67; average age 46 | NR | Open to professionals in the human service sector; programme described as affordable. | NR | No |
| **Jones et al. (2025)** | USA; online | 56% was white, 35% Asian, 9% underrepresented race and ethnicity in medicine) included 3 individuals who indicated they were Hispanic/Latino. | Faculty K-award recipients (career-development awardees); 62% associate professors, 68% held national leadership positions | Women | MD 20 (59%); MD/PhD 5 (15%); non-MD doctoral 9 (26%) | NR | One participant said that their peer group had “maybe some religious diversity,” but no religions or percentages were identified; Institutions nominated small numbers of participants for the EMPOWER pilot | Sexual orientation was 100% heterosexual/straight 100%; one participant said that their peer group had “maybe some religious diversity,” but no religions or percentages were identified | 95% were married or domestically partnered; 100% had children with 56% requiring adult supervision | Institutions nominated small numbers of participants for the EMPOWER pilot; NR. | NR | No |
| **Jowett et al. (2024)** | United Kingdom; in person | NR | Coaches with 3-20 years of coaching experience | Women | Majority held a university degree (as reported) | NR | Criteria were (a) ~10 years' coaching experience and (b) prospect to coach at Olympic/Paralympic championships | Age 25-58 (M = 37.63) | NR | Nominated coaches who then applied to UK Sport; | NR | No |
| **Kang et al. (2024)** | USA; internal then online during pandemic | White 26 (63.4%); Asian 11 (26.8%); Black 2 (4.9%); Middle Eastern or North African 1 (2.4%); Other 1 (2.4%) | Physicians, faculty and advanced practice practitioners | Women | NR | NR | Prior leadership experience 61.0% and previous leadership-programme participation 57.5% | Age 31-40 36.6%, 41-50 31.7%, 51-60 24.4%, ≥60 4.9% | caregiver in personal life: yes 15 (37.5%) | NR. | Mayo Clinic Rochester Department of Medicine; Division of General Internal Medicine + Division of Gastroenterology and Hepatology | No |
| **Kelly et al. (2021)** | USA; monthly virtual and in-person meetings | White 46%; Black 31%; Asian American 8%; Native Hawaiian/Other Pacific Islander 8%; White and Asian American 8% | Early- to mid-career physicians and scientists; early career (1-10 years post-training) 54%, mid-career (11-20 years) 46%; worked full-time (at least 40 hours/week) | Women | Medical degree required; MD or DO inferred from professional role. | Worked full-time (≥40 hours/week); partner medical associations provided protected time | Selected through competitive processes or appointment by partner associations; protected participation time provided by partner associations | Median age 42; sexual orientation: heterosexual 12 (92%), prefer not to answer 1 (8%) | Motherhood and desired workplace flexibility discussed | Selected through competitive processes or appointment by partner associations; partner organisations were asked to intentionally attend to diversity across age, race/ethnicity, year in practice, geographic location and position type; protected participation time provided by partner associations. | Physicians Foundation (supported WEL including programme evaluation); partner medical associations provided protected time | Yes |
| **Knipfer et al. (2017)** | Germany; in person | NR | Scientists: postdoctoral researchers, and assistant professors; part-time and/or short-term contracts noted as a structural barrier | Women | NR | Part-time and/or short-term contracts noted as a structural barrier | NR | NR | NR | NR. | NR | No |
| **Kvach et al. (2017)** | USA and Ethiopia; residential | NR (Ethiopian faculty; nationality reported) | Ethiopian medical faculty, aged 20s-40s | Women | NR (medical faculty; degrees not explicitly reported) | Programme duration limited to minimise time away from work | Competitive fellowship selection | Age 20s-40s | NR | Competitive fellowship selection; programme duration limited to minimise time away from work. | Funded by MEPI grant awarded by the US government; AAU-SoM and Hawassa and Haramaya University partnership | Yes |
| **Lämsä & Savela (2019)** | Finland; hybrid | NR | Mid-career professionals with approximately 20 years’ work experience and 12 years in leadership; most represented the private sector | Women | MBA participants | 10 owned their own businesses; tuition fees applied but were substantially lower than comparable MBAs; EU Social Fund financed most costs and participants paid additional tuition | NR | Ages 35-54 (mean 42) | NR | Recruitment NR; tuition fees applied but were substantially lower than comparable MBAs; flexible modular delivery supported work-family balance. | EU Social Fund financed most costs and participants paid additional tuition. | No |
| **Lee et al. (2024)** | USA; hybrid | White 32 (68%); Asian 6 (13%); Black or African American 1 (2%); American Indian or Alaska Native 4 (9%); Mixed 2 (4%); prefer not to answer 2 (4%); not Hispanic 43 (91%); 17% were underrepresented in medicine by race/ethnicity | Physicians and scientists; early-career faculty; underrepresented in medicine by race/ethnicity 17%; 42% held a recognised leadership position during the programme; mean years at rank 1.6 | Women | MD/DO 58% | NR | Self-nomination or nomination followed by selection | NR | NR | Self-nomination or nomination followed by selection; NR. | Institutionally delivered (JHUSOM) | No |
| **Martínez-Martínez et al. (2021)** | Spain; in person | NR | Executives from diverse industries; promoted at least 1 year post-programme; average organisational tenure 13 years | Women | NR | NR | Selected from women managers with ≥15 years' professional experience and company support; organisational support was required | Women’s ages reported (multinational corporate context) | NR | Selected from women managers with ≥15 years' professional experience and company support; organisational support was required. | Promociona Project launched by CEOE + Spanish Ministry of Gender Equality | Yes |
| **Mbilishaka et al. (2023)** | USA; online | Black women faculty (all participants) | Assistant professors of psychology, aged 33-37 years (mean 34); completed PhDs | Women | PhD in psychology (all); doctoral degrees from a historically Black college or university (as described) | NR | Invited and selected | Age 33-37 (mean 34) | Roles of mother and caregiver described as creating complexity for availability | Invited and selected; NR. | NR | No |
| **Megheirkouni & Roomi (2017)** | United Kingdom; in person | NR | Leaders in middle or senior management roles across several sports organisations | Women | NR | NR | Selected | NR | NR | Selected; access support NR. | UK Sport, British Olympic Foundation and Central Council of Physical Recreation | No |
| **Miles-Cohen et al. (2020)** | USA; in person | White/Caucasian (n = 230), 105 were classified as women of colour. Among the reported racial and ethnic groups, Black/African American participants were the largest group (n = 38), Asian (n = 27) and Hispanic/Latino participants (n = 27). Smaller numbers identified as mixed race (n = 10), Arab American (n = 2), Native American (n = 1), or other (n = 2); two participants did not report race or ethnicity. | Mid-career and senior/advanced psychologists; 10-20 years or more than 20 years post-doctorate | Women | Doctoral degree (all; psychologists) | Registration fees applied with scholarships available through the American Psychological Foundation; APA-funded with APF scholarship support | NR | 28 LGBTQ participants and 14 participants with disability status | NR | Competitive national application process; registration fees applied with scholarships available through the American Psychological Foundation. | APA-funded with APF scholarship support | No |
| **Mohanakumar et al. (2022)** | Asia-Pacific region; online | NR (multinational Asia-Pacific cohort; nationalities by administration) | Customs officers from 11 customs administrations | Women | NR (individual author profiles report degrees; cohort-level attainment NR) | NR | Selected via administrative processes | NR | NR | Selected via administrative processes; NR | NR | No |
| **Nakamura et al. (2022)** | Australia, India, Austria, and the United States; in-person | NR | Professionals with more than 5 years of management experience across chemicals, pharmaceuticals, higher education, manufacturing, media and R&D | Women | NR | NR | NR | NR | NR | NR. | NR | No |
| **Nash & Moore (2018)** | Antarctica (Australia run); hybrid including residential (voyage-based) | All white | STEMM professionals from five industrialised countries; most in full-time, paid, skilled employment | Women | All tertiary educated | Most in full-time, paid, skilled employment; most were in full-time, skilled employment and described as occupationally middle class; participant cost AUD$22,500; Participant fees | NR | Age 24-60 (mean 39); career stage ranged from PhD candidate to professor; 21 of 25 were married or partnered and 22 of 25 identified as heterosexual | 21 of 25 were married or partnered and 22 of 25 identified as heterosexual | Recruitment NR; participant cost AUD$22,500. | Participant fees; programme partners/sponsors | No |
| **Nash & Moore (2021)** | Antarctica (Australia run); hybrid including residential | All white | STEMM professionals from five industrialised countries; most in full-time, paid, skilled employment and described as occupationally middle class | Women | All tertiary educated | Most in full-time, paid skilled employment (occupationally middle-class, as described); participant cost AUD$25,000 | NR | Age 24-60 (mean 39); 17 of 19 married/partnered and heterosexual; 10 had children at home aged 18 months-17 years; career stage ranged from PhD candidate to professor | 17 of 19 married/partnered and heterosexual; 10 had children at home aged 18 months-17 years | Recruitment NR; participant cost AUD$25,000. | As above | No |
| **Nowling et al. (2018)** | USA; in person | NR | Faculty, mostly instructors or assistant professors (early-career focus) | Women | NR | Now relies on faculty volunteers with direct costs ~$2,300 | Self-nomination or faculty nomination followed by selection | NR | NR | Self-nomination or faculty nomination followed by selection; NR | Initially ~$10,000-$12,000 including travel/speakers; now relies on faculty volunteers with direct costs ~$2,300 | Yes |
| **O’Brien & Allin (2022)** | United Kingdom; residential | Predominantly White and degree-educated (as described) | Outdoor-sector workers or former workers seeking career progression | Women | Predominantly degree-educated | NR | Application process and selection day | Aged 23-33 | NR | Application process and selection day; NR. | Outward Bound (provider-delivered) | Yes |
| **Parker et al. (2018)** | Australia; in person; 42% were born overseas | 58% born in Australia | Academics at senior lecturer level seeking promotion to associate professor | Women | 90% held a PhD. | 56% had taken a career break and 8% had interrupted employment to support a spouse | Written application and head-of-school support required; selection based on application and commitment to attend all modules | NR | 78% had children, including 18% with three or more; 7% had caring responsibilities; 56% had taken a career break and 8% had interrupted employment to support a spouse; 72% married | Written application and head-of-school support required; selection based on application and commitment to attend all modules; NR. | Institutionally supported by the university | Yes |
| **Pelfrey et al. (2022)** | USA; in person | Caucasian 68%, Asian/Pacific Islander 20%, Hispanic/Latina 8%, other 4%. | Faculty in academic medicine and health sciences; 100% were not tenure-tracked; 80% had worked at the institution for more than 6 years | Women | 76% had a MD, 12% had a PhD | NR | Applied or nominated then selected based on leadership readiness | NR | NR | Applied or nominated then selected based on leadership readiness; NR | Institutional programme; study supported by two grants totalling $6,750. US$2,500 participation fee. | No |
| **Peterson (2019)** | Sweden; in person | NR | Senior academics and academic leaders; all were rectors | Women | NR | NR | NR | Between 52 and 67 years old, and they had between 20 and 30 years of experience | NR | Open to academic women in research and teaching positions; NR. | NR | Yes |
| **Round et al. (2024)** | Japan and Australia; online | Approximately two-thirds responded, with participants from Australia, Japan, France, the USA and Vietnam. | Leaders and managers across sectors, including senior leaders such as general managers; less than 7 years of leadership experience | Women | NR | NR | Targeted invitations | Most participants were aged between 31 and 50 | NR | Targeted invitations; NR. | NR | No |
| **Russell et al. (2023)** | USA; in person | Reports 76.9% White and 23.1% non-White | Association members developing as leaders | Women | NR | NR | Association members developing as leaders | Age 20-50+ years: 20-29 years 23.1%, 30-39 years 34.6%, 40-49 years 23.1%, and ≥50 years 19.2%. | NR | NR | Association internal funding with participation was free. | No |
| **Ryan et al. (2021)** | Ireland; in person | NR | Healthcare professionals and managers developing leader identity; all held organisational leadership roles, defined as having two or more direct reports | Women | NR | NR | Invited then applied | Average age 43, Average of 12 years of service in the organisation and, on average, over three years in a leadership role | NR | Invited then applied; NR. | NR | No |
| **Schwartz et al. (2021)** | USA; hybrid | NR | Paediatric anaesthesiologists; protégés and advisors | Women | Medical degree required; MD or DO inferred from professional role. | Women are the minority in full-time academic anaesthesiology (they comprise 41%) | Protégés and advisors; Protégés initially nominated by chief/chair, later expanded to self-nomination or nomination by other leaders (CV and personal statement); advisors nominated on experience and skills and matched by the advisory board | NR | NR | Protégés initially nominated by chief/chair, later expanded to self-nomination or nomination by other leaders (CV and personal statement); advisors nominated on experience and skills and matched by the advisory board. | Society for Pediatric Anesthesia and Masimo. Protégés paid US$450/year; advisors and GAIN networking participated free of charge. | No |
| **Sears et al. (2019)** | USA; in-person events; intranet-based networking | NR | Physicians across employment models and career stages | Women | Medical degree required; MD or DO inferred from professional role. | Release time was rarely provided | NR | NR | NR | Available to all employed or affiliated BSWH physicians; release time was rarely provided. | BSWH board of directors funded the WLiM pilot internally | No |
| **Sears et al. (2025)** | USA; in-person events; intranet-based networking | White/non-Hispanic 60.9%, Asian 19.6%, Black/African American 8.0%, Hispanic/Latina 5.8%, multiple/mixed race 2.2%, other race 2.9%, Middle Eastern 0.7%. | Physicians with at least 5 years of clinical experience | Women | Medical degree required; MD or DO inferred from professional role. | 74.8% worked 1.0 FTE | NR | Median age 41 (IQR 37,48); 26.5% had children aged <5 years and 46.4% had school-aged children | Married/partnered 84.7%; 26.5% had children aged <5 years and 46.4% had school-aged children | Available to all employed or affiliated BSWH physicians; CME time could be used to attend. | The Physicians Foundation grant funding ($150,000); health system support | No |
| **Selzer et al. (2017)** | USA; in person | 66.6% White; 33.3% African American | Higher education faculty and administrators; all had at least 3 years of university employment | Women | All had doctoral degrees | Applicants' home departments invest $100 in financial support | Invitation, formal application and competitive selection | Age (all in our forties) | motherhood (all have children); married to men (one now divorced) | Invitation, formal application and competitive selection; NR | University programme; Applicants' home departments invest $100 in financial support | Yes |
| **Sims et al. (2022)** | USA; in person | White 79% | Tenured faculty; 43% were associate professors* | Mixed-gender cohort; women 71%, men 29% | Doctorate 93%* | Very few received course release and most participated in their own time | Competitive selection by the ADVANCE Executive Leadership Committee | Largest group aged 40-49 (57%)* | Married 79%; 72% had children at home: reported* | Competitive selection by the ADVANCE Executive Leadership Committee; very few received course release and most participated in their own time. | NSF ADVANCE grant | Yes |
| **Skarupski et al. (2019)** | USA; in person | None collected | Faculty from assistant to full professor with at least 4 years at the institution; 42 graduates (15%) were known to have left the institution | Women | None collected | NR | NR | NR | NR | NR. | NR | No |
| **Spalluto et al. (2017)** | USA; in person | NR | Radiologists, including faculty and trainees; 47.1% were assistant professors and 17.6% professors; academic experience ranged from 0 to more than 20 years, with 53.0% having 6-15 years’ experience | Women | NR | 82.4% held full-time appointments; Funding in conjunction with financial support from the department chair was obtained to fund the programme | NR | Age 30-60+ years; most were aged 35-44 (53.0%) | NR | All women faculty on clinical and research tracks within the radiology department were eligible; NR. | Funding in conjunction with financial support from the department chair was obtained to fund the programme | No |
| **Sutton et al. (2024)** | USA; hybrid | NR | Postdoctoral research fellows performing cancer research; most were 1-7 years post-PhD | Women | Highest degree (typically PhD) | One-time recruitment stipend provided | Competitively selected from self-nominated applications | Life events (pregnancies, births, marriages, divorces) noted as accommodated (programme flexibility) | Life events (pregnancies, births, marriages, divorces) noted as accommodated (programme flexibility) | Competitively selected from self-nominated applications; one-time recruitment stipend provided; NR. | Funded through the estate of Sallie Rosen Kaplan (endowment) via the NCI intramural programme | No |
| **Valerio et al. (2024)** | Asia-Pacific region (Southeast Asia and the Pacific); in person in Australia | NR (countries across Southeast Asia and the Pacific) | Agricultural researchers; 11 employed by governmental research institutions | Women | Doctorate 8 (53%); over half held doctorates | NR | Open call with selection based on leadership experience, aspirations and panel rankings | NR | Work–family/caregiving discussed qualitatively (e.g. elderly-parent care, childcare); no participant count given | Open call with selection based on leadership experience, aspirations and panel rankings; NR. | NR | No |
| **Van Oosten et al. (2017)** | USA; in person | NR | STEM technical professionals and managers with 5-25 years of work experience | Women | NR | NR | Nominated by organisation; organisational support provided | NR | NR (a case vignette describes a composite participant as married with three children, but no cohort-level demographic data are reported) | Nominated by organisation; organisational support provided. | Employer sponsorship; university executive education partnership | No |
| **Walsh et al. (2022)** | USA & LMICs; virtual/remote components | No explicit race/ethnicity, but nationality/culture is reported: 10 researchers from 6 countries (Haiti, India, Kenya, Tanzania, Uganda, and the United States) | Early-career researchers in global health | Women | NR | Participants received a US$500 stipend and travel support for the symposium and workshop; described as novel low-cost intervention | Application and selection | NR | NR | Application and selection; participants received a US$500 stipend and travel support for the symposium and workshop. | Institutionally supported; described as novel low-cost intervention | Yes |

***Note*.** NR = not reported in the source paper after full-text review. Unclear = reported ambiguously; basis stated in cell or in Queries for review. Author terminology preserved for race, ethnicity, gender, disability, sexual orientation, career stage and roles. Accessibility codes: Open enrolment; Competitive selection; Invitation only; Nomination; Supervisor endorsement required (a programme may carry more than one code); the study-reported access description follows the codes. Participant-facing costs are distinguished from programme funding. No characteristic was inferred from occupation, institution or location. Demographic characteristics were reported for the overall mixed-gender participant cohort rather than separately for women and men.

# Supplementary Table S6. Study-level outcomes for women mapped to the extended Kirkpatrick framework

| **Study** | **1: Reaction** | **2A: Knowledge (subjective)** | **2B: Knowledge (objective)** | **3A: Behaviour/ expertise (subjective)** | **3B: Behaviour/ expertise (objective)** | **4A: System results/ performance (subjective)** | **4B: System results/ performance (objective)** |
| --- | --- | --- | --- | --- | --- | --- | --- |
| **Adeola, 2024** | x | x |  | x | x | x |  |
| **Barnard, 2022** | x | x | x | x |  | x |  |
| **Brixey, 2025** | x | x |  |  |  |  |  |
| **Brue, 2016** | x | x |  | x | x | x | x |
| **Brue, 2018** | x | x |  | x | x |  |  |
| **Chang, 2016** |  |  |  |  | x |  | x |
| **Chang, 2020** |  |  |  |  | x |  | x |
| **Chasserio, 2024** |  | x |  | x | x | x | x |
| **Chaudron, 2021** |  |  | x | x |  |  | x |
| **Cordeiro, 2024** |  | x |  | x | x |  | x |
| **Danhauer, 2019** | x | x | x | x |  |  | x |
| **David, 2025** | x | x |  | x | x |  | x |
| **DeFrank-Cole, 2017** | x | x | x | x |  |  | x |
| **Deutsch, 2022** | x | x |  | x | x |  |  |
| **Drake, 2023** |  |  | x |  | x |  | x |
| **Fassiotto, 2024** | x | x |  | x | x |  | x |
| **Ford, 2021** |  |  | x |  | x |  |  |
| **Grando, 2022** | x | x | x |  | x |  |  |
| **Heilemann, 2024** |  | x |  | x |  |  |  |
| **Helitzer, 2016** | x | x |  | x | x | x |  |
| **Herbst, 2024** | x | x | x | x |  |  |  |
| **Hopkins, 2022** |  | x | x | x | x | x | x |
| **Jones, 2025** | x | x |  | x |  |  |  |
| **Jowett, 2024** | x | x |  | x | x | x | x |
| **Kang, 2024** | x | x | x |  |  |  |  |
| **Kelly, 2021** | x | x |  | x |  |  |  |
| **Knipfer, 2017** | x | x | x | x |  |  |  |
| **Kvach, 2017** | x | x |  | x |  | x |  |
| **Lämsä, 2019** | x | x |  | x | x | x | x |
| **Lee, 2024** | x | x | x | x |  |  |  |
| **Martínez-Martínez, 2021** | x | x |  | x |  |  | x |
| **Mbilishaka, 2023** |  | x |  |  |  |  |  |
| **Megheirkouni, 2017** | x | x |  | x |  |  |  |
| **Miles-Cohen, 2020** | x |  |  | x | x | x |  |
| **Mohanakumar, 2022** | x | x |  | x |  |  |  |
| **Nakamura, 2022** | x | x |  |  |  |  |  |
| **Nash, 2018** | x | x |  |  |  |  |  |
| **Nash, 2021** |  | x |  | x |  |  | x |
| **Nowling, 2018** | x | x |  |  | x |  | x |
| **O’Brien, 2022** |  | x |  |  |  |  |  |
| **Parker, 2018** | x | x |  | x |  |  |  |
| **Pelfrey, 2022** | x | x |  | x | x |  |  |
| **Peterson, 2019** | x | x |  | x |  | x | x |
| **Round, 2024** | x | x | x |  |  |  |  |
| **Russell, 2023** | x | x |  | x | x | x | x |
| **Ryan, 2021** |  | x |  | x |  |  |  |
| **Schwartz, 2021** | x | x |  | x | x |  | x |
| **Sears, 2019** | x | x |  |  |  | x |  |
| **Sears, 2025** |  |  | x |  |  |  | x |
| **Selzer, 2017** | x | x |  |  |  |  |  |
| **Sims, 2022*** |  | x | x |  |  |  |  |
| **Skarupski, 2019.** |  | x |  |  |  |  |  |
| **Spalluto, 2017** | x | x | x |  | x |  | x |
| **Sutton, 2024** | x | x | x | x |  |  | x |
| **Valerio, 2024** |  | x |  | x |  | x |  |
| **Van Oosten, 2017** | x | x |  | x | x | x | x |
| **Walsh, 2022** | x | x |  |  | x |  | x |
| **Total, n (%)** | **40 (70%)** | **50 (88%)** | **17 (30%)** | **37 (65%)** | **25 (44%)** | **15 (26%)** | **25 (44%)** |

***Note***. x = outcome reported at this level. 2A/3A/4A = subjective (self-reported or perceived); 2B/3B/4B = objective (concrete/tangible outcomes, whether externally verified or reported by participants). Cells indicate the extended Kirkpatrick level(s) at which each study reported an outcome for women. Outcomes were coded only where attributable to women. Where a programme also enrolled men and an outcome was reported for the combined cohort without disaggregation by sex, that outcome was not mapped; this most often affects higher-level behavioural (Level 3) and organisational (Level 4) results. Consequently, mixed-sex programmes may display fewer coded cells than the full range of outcomes they report.

Supplementary Tables S7A-S7F. Sensitivity and exploratory analyses

# Supplementary Table S7A. Sensitivity analysis by presence of a pre-programme baseline

| Extended Kirkpatrick level | Pre-programme baseline (n=23) | No pre-programme baseline (n=34) | All studies (n=57) |
| --- | --- | --- | --- |
| Level 1: Reaction | 16/23 (70%) | 24/34 (71%) | 40/57 (70%) |
| Level 2A: Knowledge (subjective) | 18/23 (78%) | 32/34 (94%) | 50/57 (88%) |
| Level 2B: Knowledge (objective) | 16/23 (70%) | 1/34 (3%) | 17/57 (30%) |
| Level 3A: Behaviour (subjective) | 11/23 (48%) | 26/34 (76%) | 37/57 (65%) |
| Level 3B: Behaviour (objective) | 10/23 (43%) | 15/34 (44%) | 25/57 (44%) |
| Level 4A: System results (subjective) | 3/23 (13%) | 12/34 (35%) | 15/57 (26%) |
| Level 4B: System results (objective) | 12/23 (52%) | 13/34 (38%) | 25/57 (44%) |
| Any Level 4 (4A and/or 4B) | 13/23 (57%) | 19/34 (56%) | 32/57 (56%) |
| Any objective Level 3B or 4B | 15/23 (65%) | 18/34 (53%) | 33/57 (58%) |

**Pre-programme baseline (n=23):** Barnard, 2022; Brixey, 2025; Chang, 2020; Chasserio, 2024; Chaudron, 2021; Danhauer, 2019; DeFrank-Cole, 2017; Drake, 2023; Ford, 2021; Grando, 2022; Herbst, 2024; Kang, 2024; Knipfer, 2017; Lämsä, 2019; Lee, 2024; Nowling, 2018; Pelfrey, 2022; Round, 2024; Sears, 2025; Sims, 2022; Spalluto, 2017; Sutton, 2024; Walsh, 2022.

**No pre-programme baseline (n=34):** Adeola, 2024; Brue, 2016; Brue, 2018; Chang, 2016; Cordeiro, 2024; David, 2025; Deutsch, 2022; Fassiotto, 2024; Heilemann, 2024; Helitzer, 2016; Hopkins, 2022; Jones, 2025; Jowett, 2024; Kelly, 2021; Kvach, 2017; Martínez-Martínez, 2021; Mbilishaka, 2023; Megheirkouni, 2017; Miles-Cohen, 2020; Mohanakumar, 2022; Nakamura, 2022; Nash, 2018; Nash, 2021; O’Brien, 2022; Parker, 2018; Peterson, 2019; Russell, 2023; Ryan, 2021; Schwartz, 2021; Sears, 2019; Selzer, 2017; Skarupski, 2019; Valerio, 2024; Van Oosten, 2017.

***Note.*** Baseline was defined as programme outcome or relevant participant-status data available at or before programme commencement (“Pre”, Table S3), including prospectively collected baseline measures and clearly defined administrative or registry baseline data. Post-only, mid-only, follow-up-only and retrospective studies without a defined pre-programme participant baseline were classified as having no baseline. Archival pre-programme materials used only for contextual interpretation, rather than as baseline outcome data, were not counted as a baseline.

# Supplementary Table S7B. Sensitivity analysis by presence of a non-participant comparison group

| **Extended Kirkpatrick level** | **Comparison group (n=4)** | **No comparison group (n=53)** | **All studies (n=57)** |
| --- | --- | --- | --- |
| Level 1: Reaction | 1/4 | 39/53 | 40/57 |
| Level 2A: Knowledge (subjective) | 1/4 | 49/53 | 50/57 |
| Level 2B: Knowledge (objective) | 2/4 | 15/53 | 17/57 |
| Level 3A: Behaviour (subjective) | 1/4 | 36/53 | 37/57 |
| Level 3B: Behaviour (objective) | 2/4 | 23/53 | 25/57 |
| Level 4A: System results (subjective) | 1/4 | 14/53 | 15/57 |
| Level 4B: System results (objective) | 3/4 | 22/53 | 25/57 |
| Any Level 4 (4A and/or 4B) | 4/4 | 28/53 | 32/57 |
| Any objective Level 3B or 4B | 3/4 | 30/53 | 33/57 |

**Comparison group (n=4):** Barnard, 2022; Chang, 2016; Chang, 2020; Sears, 2025

***Note.*** Comparison group = a verified non-participant comparator: Chang 2016 and Chang 2020 included non-participant women faculty drawn from the AAMC Faculty Roster; Barnard 2022 included non-Aurora women recruited through snowball sampling; and Sears 2025 included internal and external non-participant physician comparison groups. Within-participant pre-post comparisons and comparisons between programme cohorts were not counted as non-participant comparison groups. Percentages are omitted because n=4 and each study changes the percentage by 25 percentage points.

# Supplementary Table S7C. Sensitivity analysis by risk of bias

| **Extended Kirkpatrick level** | **Low risk of bias (n=32)** | **Moderate or high risk of bias (n=25)** | **All studies (n=57)** |
| --- | --- | --- | --- |
| Level 1: Reaction | 21/32 (66%) | 19/25 (76%) | 40/57 (70%) |
| Level 2A: Knowledge (subjective) | 29/32 (91%) | 21/25 (84%) | 50/57 (88%) |
| Level 2B: Knowledge (objective) | 3/32 (9%) | 14/25 (56%) | 17/57 (30%) |
| Level 3A: Behaviour (subjective) | 24/32 (75%) | 13/25 (52%) | 37/57 (65%) |
| Level 3B: Behaviour (objective) | 14/32 (44%) | 11/25 (44%) | 25/57 (44%) |
| Level 4A: System results (subjective) | 9/32 (28%) | 6/25 (24%) | 15/57 (26%) |
| Level 4B: System results (objective) | 13/32 (41%) | 12/25 (48%) | 25/57 (44%) |
| Any Level 4 (4A and/or 4B) | 16/32 (50%) | 16/25 (64%) | 32/57 (56%) |
| Any objective Level 3B or 4B | 17/32 (53%) | 16/25 (64%) | 33/57 (58%) |

**Low risk of bias (n=32):** Brue, 2016; Brue, 2018; Chang, 2016; Chang, 2020; Chasserio, 2024; Cordeiro, 2024; David, 2025; Deutsch, 2022; Heilemann, 2024; Helitzer, 2016; Herbst, 2024; Jones, 2025; Jowett, 2024; Kelly, 2021; Kvach, 2017; Lee, 2024; Mbilishaka, 2023; Megheirkouni, 2017; Mohanakumar, 2022; Nakamura, 2022; Nash, 2018; Nash, 2021; O’Brien, 2022; Pelfrey, 2022; Peterson, 2019; Russell, 2023; Ryan, 2021; Schwartz, 2021; Sears, 2025; Selzer, 2017; Valerio, 2024; Van Oosten, 2017.

**Moderate or high risk of bias (n=25):** Adeola, 2024; Barnard, 2022; Brixey, 2025; Chaudron, 2021; Danhauer, 2019; DeFrank-Cole, 2017; Drake, 2023; Fassiotto, 2024; Ford, 2021; Grando, 2022; Hopkins, 2022; Kang, 2024; Knipfer, 2017; Lämsä, 2019; Martínez-Martínez, 2021; Miles-Cohen, 2020; Nowling, 2018; Parker, 2018; Round, 2024; Sears, 2019; Sims, 2022; Skarupski, 2019; Spalluto, 2017; Sutton, 2024; Walsh, 2022.

***Note.*** Groups use the final JBI overall appraisal ratings reported in Supplementary Tables S6A-S6F: low risk = ≥70% of applicable items scored “yes”; moderate risk = 50–69%; and high risk = ≤49%. Items rated N/A were excluded from the denominator. Objective Level 2B outcomes were more frequently reported among studies rated moderate or high risk than among studies rated low risk. Reporting of Level 3B outcomes was equivalent across the two strata, while Level 4B and the combined objective Level 3B or 4B indicator were somewhat more frequent in the moderate- or high-risk group. These patterns partly reflect the concentration of single-group pre-post assessments and administrative outcome designs within the moderate- or high-risk group.

# Supplementary Table S7D. Sensitivity analysis by long-term follow-up

| **Extended Kirkpatrick level** | **Follow-up ≥1 year (n=16)** | **No follow-up ≥1 year (n=41)** | **All studies (n=57)** |
| --- | --- | --- | --- |
| Level 1: Reaction | 10/16 (63%) | 30/41 (73%) | 40/57 (70%) |
| Level 2A: Knowledge (subjective) | 11/16 (69%) | 39/41 (95%) | 50/57 (88%) |
| Level 2B: Knowledge (objective) | 3/16 (19%) | 14/41 (34%) | 17/57 (30%) |
| Level 3A: Behaviour (subjective) | 11/16 (69%) | 26/41 (63%) | 37/57 (65%) |
| Level 3B: Behaviour (objective) | 10/16 (63%) | 15/41 (37%) | 25/57 (44%) |
| Level 4A: System results (subjective) | 6/16 (38%) | 9/41 (22%) | 15/57 (26%) |
| Level 4B: System results (objective) | 10/16 (63%) | 15/41 (37%) | 25/57 (44%) |
| Any Level 4 (4A and/or 4B) | 13/16 (81%) | 19/41 (46%) | 32/57 (56%) |
| Any objective Level 3B or 4B | 14/16 (88%) | 19/41 (46%) | 33/57 (58%) |

**Follow-up ≥1 year (n=16):** Barnard, 2022; Chang, 2016; Chang, 2020; Chaudron, 2021; Fassiotto, 2024; Ford, 2021; Helitzer, 2016; Jowett, 2024; Lämsä, 2019; Martínez-Martínez, 2021; Miles-Cohen, 2020; Nash, 2021; Nowling, 2018; Pelfrey, 2022; Peterson, 2019; Skarupski, 2019.

**No follow-up ≥1 year (n=41):** Adeola, 2024; Brixey, 2025; Brue, 2016; Brue, 2018; Chasserio, 2024; Cordeiro, 2024; Danhauer, 2019; David, 2025; DeFrank-Cole, 2017; Deutsch, 2022; Drake, 2023; Grando, 2022; Heilemann, 2024; Herbst, 2024; Hopkins, 2022; Jones, 2025; Kang, 2024; Kelly, 2021; Knipfer, 2017; Kvach, 2017; Lee, 2024; Mbilishaka, 2023; Megheirkouni, 2017; Mohanakumar, 2022; Nakamura, 2022; Nash, 2018; O’Brien, 2022; Parker, 2018; Round, 2024; Russell, 2023; Ryan, 2021; Schwartz, 2021; Sears, 2019; Sears, 2025; Selzer, 2017; Sims, 2022; Spalluto, 2017; Sutton, 2024; Valerio, 2024; Van Oosten, 2017; Walsh, 2022.

***Note.*** Long-term follow-up was defined as at least one programme outcome assessed ≥12 months after a defined pre-programme baseline or programme completion (“Post-long”, Table S3). Programme duration alone did not qualify. Retrospective studies were included where programme outcomes were assessed among former participants at intervals extending to ≥12 months after programme participation. Helitzer 2016 was therefore included because its retrospective alumnae evaluation encompassed programme participation extending many years before the 2011 assessment. Fassiotto 2024, which administered annual surveys through Year 5; Lämsä 2019, which included assessment approximately 18 months after programme completion; and Martínez-Martínez 2021, in which interviews occurred 1–6 years after the Promociona programme, were also included. Deutsch 2022 was excluded because its 6- and 14-month follow-up waves were anchored to an initial survey conducted after WELI had already commenced rather than to a clearly defined pre-programme baseline or programme completion. Sears 2019 (six-month follow-up), Heilemann 2024 (1-, 4- and 9-month assessments) and Spalluto 2017 (end-of-Year-1 formative evaluation during a two-year programme) were excluded. Schwartz 2021 was excluded because it used a single cross-sectional survey of an ongoing programme with variable participant exposure and no common ≥12-month post-baseline or post-completion follow-up point.

# Supplementary Table S7E. Exploratory analysis by reported organisational embedding and support

| **Extended Kirkpatrick level** | **Reported organisational embedding/support (n=40)** | **No reported organisational embedding/support (n=17)** | **All studies (n=57)** |
| --- | --- | --- | --- |
| Level 1: Reaction | 28/40 (70%) | 12/17 (71%) | 40/57 (70%) |
| Level 2A: Knowledge (subjective) | 33/40 (83%) | 17/17 (100%) | 50/57 (88%) |
| Level 2B: Knowledge (objective) | 14/40 (35%) | 3/17 (18%) | 17/57 (30%) |
| Level 3A: Behaviour (subjective) | 25/40 (63%) | 12/17 (71%) | 37/57 (65%) |
| Level 3B: Behaviour (objective) | 18/40 (45%) | 7/17 (41%) | 25/57 (44%) |
| Level 4A: System results (subjective) | 10/40 (25%) | 5/17 (29%) | 15/57 (26%) |
| Level 4B: System results (objective) | 19/40 (48%) | 6/17 (35%) | 25/57 (44%) |
| Any Level 4 (4A and/or 4B) | 25/40 (63%) | 7/17 (41%) | 32/57 (56%) |
| Any objective Level 3B or 4B | 24/40 (60%) | 9/17 (53%) | 33/57 (58%) |

## Additional descriptive evaluation characteristics (not Kirkpatrick levels):

| **Evaluation characteristic** | **Reported organisational embedding/support (n=40)** | **No reported organisational embedding/support (n=17)** | **All studies (n=57)** |
| --- | --- | --- | --- |
| Used administrative or externally verifiable records | 12/40 (30%) | 0/17 (0%) | 12/57 (21%) |
| Long-term follow-up ≥1 year | 12/40 (30%) | 4/17 (24%) | 16/57 (28%) |
| Non-participant comparison group | 4/40 (10%) | 0/17 (0%) | 4/57 (7%) |
| Low risk of bias | 20/40 (50%) | 12/17 (71%) | 32/57 (56%) |

**Reported organisational embedding/support (n=40):** Barnard, 2022; Brixey, 2025; Chang, 2016; Chang, 2020; Chaudron, 2021; Cordeiro, 2024; Danhauer, 2019; David, 2025; Deutsch, 2022; Drake, 2023; Fassiotto, 2024; Ford, 2021; Grando, 2022; Heilemann, 2024; Helitzer, 2016; Herbst, 2024; Kang, 2024; Kelly, 2021; Knipfer, 2017; Kvach, 2017; Lämsä, 2019; Lee, 2024; Martínez-Martínez, 2021; Miles-Cohen, 2020; Nowling, 2018; O’Brien, 2022; Parker, 2018; Peterson, 2019; Russell, 2023; Ryan, 2021; Schwartz, 2021; Sears, 2019; Sears, 2025; Selzer, 2017; Sims, 2022; Spalluto, 2017; Sutton, 2024; Valerio, 2024; Van Oosten, 2017; Walsh, 2022.

**No reported organisational embedding/support (n=17):** Adeola, 2024; Brue, 2016; Brue, 2018; Chasserio, 2024; DeFrank-Cole, 2017; Hopkins, 2022; Jones, 2025; Jowett, 2024; Mbilishaka, 2023; Megheirkouni, 2017; Mohanakumar, 2022; Nakamura, 2022; Nash, 2018; Nash, 2021; Pelfrey, 2022; Round, 2024; Skarupski, 2019.

***Note.*** Organisational embedding/support = an explicit report of at least one of the following: senior-leadership sponsorship; protected time; dedicated funding, staffing or in-kind support; access to institutional HR, promotion or retention data; or integration within a broader gender-equity initiative. Classification was based on explicit reporting in the programme-characteristics and PROGRESS-Plus tables. Absence of a statement was classified as “no reported organisational embedding/support” rather than confirmed absence of support. This analysis was exploratory, and differences are reported descriptively without causal interpretation.

# ****Supplementary Table S7F. Sensitivity analysis restricted to studies with pre-post outcome measurement or a verified non-participant comparison group****

| **Extended Kirkpatrick level** | **Pre-post or controlled studies (n=24)** | **All studies (n=57)** |
| --- | --- | --- |
| Level 1: Reaction | 16/24 (67%) | 40/57 (70%) |
| Level 2A: Knowledge (subjective) | 18/24 (75%) | 50/57 (88%) |
| Level 2B: Knowledge (objective) | 16/24 (67%) | 17/57 (30%) |
| Level 3A: Behaviour (subjective) | 11/24 (46%) | 37/57 (65%) |
| Level 3B: Behaviour (objective) | 11/24 (46%) | 25/57 (44%) |
| Level 4A: System results (subjective) | 3/24 (13%) | 15/57 (26%) |
| Level 4B: System results (objective) | 13/24 (54%) | 25/57 (44%) |
| Any Level 4 | 14/24 (58%) | 32/57 (56%) |
| Any objective Level 3B or 4B | 16/24 (67%) | 33/57 (58%) |

**Pre-post or controlled studies (n=24):** Barnard, 2022; Brixey, 2025; Chang, 2016; Chang, 2020; Chasserio, 2024; Chaudron, 2021; Danhauer, 2019; DeFrank-Cole, 2017; Drake, 2023; Ford, 2021; Grando, 2022; Herbst, 2024; Kang, 2024; Knipfer, 2017; Lämsä, 2019; Lee, 2024; Nowling, 2018; Pelfrey, 2022; Round, 2024; Sears, 2025; Sims, 2022; Spalluto, 2017; Sutton, 2024; Walsh, 2022.

***Note.*** Pre-post or controlled studies were defined as studies that assessed programme participants at an explicit pre-programme outcome baseline and at least one post-programme timepoint within the same cohort, or included a verified non-participant comparison group. The stratum comprised 23 studies with pre-programme outcome data plus Chang 2016, which included a verified non-participant comparison group but no defined participant baseline. Restricting the synthesis to these studies increased the proportion reporting objective knowledge outcomes and modestly increased the proportion reporting objective system-level outcomes. These patterns reflect differences in evaluation design and measurement rather than programme effectiveness. All analyses were descriptive.

# Supplementary Table S8A. Quality appraisal: Qualitative studies (JBI Checklist)

| **Study** | **1. Philosophical congruity with methodology** | **2. Methodology-question congruity** | **3. Methodology-data collection congruity** | **4. Methodology-analysis congruity** | **5. Methodology-interpretation congruity** | **6. Researcher positionality stated** | **7. Researcher influence addressed** | **8. Participant voices represented** | **9. Ethical approval** | **10. Conclusions follow from data** | **Overall appraisal rating** |
| --- | --- | --- | --- | --- | --- | --- | --- | --- | --- | --- | --- |
| **Brue & Brue 2016** | Yes | Yes | Yes | Yes | Yes | Yes | Yes | Yes | Yes | Yes | Low |
| **Brue & Brue 2018** | Yes | Yes | Yes | Yes | Yes | Yes | Yes | Yes | Unclear | Yes | Low |
| **Chasserio & Bacha 2024** | Yes | Yes | Yes | Yes | Yes | No | Unclear | Yes | Unclear | Yes | Low |
| **Heilemann & Parker 2024** | Unclear | Yes | Yes | Yes | Yes | Yes | Yes | Yes | Unclear | Yes | Low |
| **Helitzer 2016** | Unclear | Yes | Yes | Yes | Yes | Yes | Unclear | Yes | Yes | Yes | Low |
| **Jones et al., 2025** | Unclear | Yes | Yes | Yes | Yes | No | Unclear | Yes | Yes | Yes | Low |
| **Jowett 2024** | Yes | Yes | Yes | Yes | Yes | Yes | Yes | Yes | Yes | Yes | Low |
| **Kvach 2017** | Unclear | Yes | Yes | Yes | Yes | No | No | Yes | Yes | Yes | Low |
| **Lämsä & Savela 2019** | Unclear | Yes | Yes | Yes | Yes | Unclear | No | Yes | Unclear | Yes | Moderate |
| **Mbilishaka 2023** | Yes | Yes | Yes | Yes | Yes | Yes | Yes | Yes | Unclear | Yes | Low |
| **Megheirkouni & Roomi 2017** | Yes | Yes | Yes | Yes | Yes | No | No | Yes | Unclear | Yes | Low |
| **Nakamura 2022** | Unclear | Yes | Yes | Yes | Yes | Yes | Unclear | Yes | Yes | Yes | Low |
| **Nash & Moore 2018** | Yes | Yes | Yes | Yes | Yes | Yes | Unclear | Yes | Yes | Yes | Low |
| **Nash & Moore 2021** | Yes | Yes | Yes | Yes | Yes | Yes | Unclear | Yes | Yes | Yes | Low |
| **O’Brien & Allin 2022** | Yes | Yes | Yes | Yes | Yes | Yes | Yes | Yes | Yes | Yes | Low |
| **Pelfrey 2022** | Yes | Yes | Yes | Yes | Yes | Unclear | Unclear | Yes | Yes | Yes | Low |
| **Peterson 2019** | Yes | Yes | Yes | Yes | Yes | Unclear | Yes | Yes | Yes | Yes | Low |
| **Ryan 2021** | Yes | Yes | Yes | Yes | Yes | Unclear | Unclear | Yes | Yes | Yes | Low |
| **Selzer 2017** | Yes | Yes | Yes | Yes | Yes | Yes | Yes | Yes | Unclear | Yes | Low |
| **Valerio, 2024** | Unclear | Yes | Yes | Yes | Yes | Unclear | Yes | Yes | Yes | Yes | Low |

***Note****.* Risk of bias was rated low (≥70% of applicable items scored ‘yes’), moderate (50-69%), or high (≤49%). N/A items were excluded from the denominator.

# Supplementary Table S8B. Quality appraisal: Quasi-experimental studies (JBI Checklist)

| **Study** | **1. Cause and effect clearly defined** | **2. Comparison groups similar** | **3. Similar co-interventions** | **4. Control group present** | **5. Multiple outcome measurements** | **6. Follow-up complete** | **7. Outcomes measured consistently** | **8. Outcomes measured reliably** | **9. Appropriate statistical analysis** | **Overall appraisal rating** |
| --- | --- | --- | --- | --- | --- | --- | --- | --- | --- | --- |
| **Kang 2024** | Yes | Yes | N/A | No | No | No | Yes | Yes | Unclear | Moderate |
| **Knipfer 2017** | Yes | Yes | N/A | No | No | No | Yes | Unclear | Unclear | High |
| **Brixey 2025** | Yes | Yes | N/A | No | No | Unclear | Yes | Unclear | Yes | Moderate |
| **Chaudron 2021** | Yes | Yes | N/A | No | No | Unclear | Yes | Unclear | Yes | Moderate |
| **Ford 2021** | Yes | Yes | N/A | No | No | Unclear | Yes | Yes | Yes | Moderate |
| **Round 2024** | Yes | Unclear | N/A | No | No | No | Yes | Yes | Unclear | High |
| **Spalluto 2017** | Yes | Unclear | N/A | No | No | Unclear | Yes | Unclear | Yes | High |
| **Walsh 2022** | Yes | Unclear | N/A | No | No | Yes | Yes | Unclear | No | High |
| **Nowling 2018** | Yes | No | No | No | No | Unclear | Yes | Yes | Yes | High |

***Note***. Risk of bias was rated low (≥70% of applicable items scored ‘yes’), moderate (50-69%), or high (≤49%). N/A items were excluded from the denominator. For single-group pre-post studies, Item 2 was interpreted as whether the pre- and post-intervention observations were derived from the same participant group; Item 4 separately assessed the presence of an independent control group.

# Supplementary Table S8C. Quality appraisal: Cross-sectional studies (JBI Checklist)

| **Study** | **1. Inclusion criteria defined** | **2. Subjects and setting described** | **3. Exposure measured validly** | **4. Standard condition criteria used** | **5. Confounders identified** | **6. Confounder strategies stated** | **7. Outcomes measured validly** | **8. Appropriate statistical analysis** | **Overall appraisal rating** |
| --- | --- | --- | --- | --- | --- | --- | --- | --- | --- |
| **Skarupski 2019** | Yes | Unclear | Yes | N/A | No | No | Yes | Yes | Moderate |
| **David, 2025** | Yes | Yes | Yes | N/A | No | No | Yes | Yes | Low |

***Note****.* Risk of bias was rated low (≥70% of applicable items scored ‘yes’), moderate (50-69%), or high (≤49%). N/A items were excluded from the denominator.

# Supplementary Table S8D. Quality appraisal: Cohort studies (JBI Checklist)

| **Study** | **1. Groups from same population** | **2. Exposures measured similarly** | **3. Exposure measured validly** | **4. Confounders identified** | **5. Confounder strategies stated** | **6. Groups free of outcome at baseline** | **7. Outcomes measured validly** | **8. Follow-up time sufficient** | **9. Follow-up complete** | **10. Strategies for incomplete follow-up** | **11. Appropriate statistical analysis** | **Overall appraisal rating** |
| --- | --- | --- | --- | --- | --- | --- | --- | --- | --- | --- | --- | --- |
| **Chang 2020** | Yes | Yes | Yes | Yes | Yes | Yes | Yes | Yes | Yes | N/A | Yes | Low |
| **Chang 2016** | Yes | Yes | Yes | Yes | Yes | Yes | Yes | Yes | Yes | N/A | Yes | Low |
| **Sutton 2024** | N/A | N/A | Yes | Unclear | No | Unclear | Yes | Yes | No | No | Unclear | High |
| **Sears 2025** | Unclear | Yes | Yes | Yes | Yes | No | Yes | Yes | Unclear | Yes | Yes | Low |
| **Fassiotto 2024** | N/A | N/A | Yes | No | No | Unclear | Unclear | Yes | Unclear | No | Unclear | High |

***Note****.* Risk of bias was rated low (≥70% of applicable items scored ‘yes’), moderate (50-69%), or high (≤49%). N/A items were excluded from the denominator. Items concerning between-group comparability were rated N/A for single-cohort studies without a separate exposed or comparison group.

# Supplementary Table S8E. Quality appraisal: Mixed-methods studies using component-specific JBI checklists

## Part 1: Studies appraised using the JBI qualitative and analytical cross-sectional checklists

| **Study** | **Q1. Philosophical congruity with methodology** | **Q2. Methodology-question congruity** | **Q3. Methodology-data collection congruity** | **Q4. Methodology-analysis congruity** | **Q5. Methodology-interpretation congruity** | **Q6. Researcher positionality stated** | **Q7. Researcher influence addressed** | **Q8. Participant voices represented** | **Q9. Ethical approval** | **Q10. Conclusions follow from data** | **CS1. Inclusion criteria defined** | **CS2. Subjects and setting described** | **CS3. Exposure measured validly** | **CS4. Standard condition criteria used** | **CS5. Confounders identified** | **CS6. Confounder strategies stated** | **CS7. Outcomes measured validly** | **CS8. Appropriate statistical analysis** | **Overall appraisal rating** |
| --- | --- | --- | --- | --- | --- | --- | --- | --- | --- | --- | --- | --- | --- | --- | --- | --- | --- | --- | --- |
| **Martínez-Martínez 2021** | Unclear | Yes | Yes | Yes | Yes | No | Unclear | Yes | Unclear | Yes | Yes | Yes | Yes | N/A | No | No | Yes | Yes | Moderate |
| **Cordeiro 2024** | Unclear | Yes | Yes | Yes | Yes | Yes | Yes | Yes | Yes | Yes | Yes | Yes | Yes | N/A | Unclear | No | Yes | Yes | Low |
| **Adeola 2024** | Unclear | Yes | Yes | Yes | Yes | No | No | Yes | Unclear | Yes | Yes | Yes | Yes | N/A | Unclear | No | No | Yes | Moderate |
| **Kelly 2021** | Unclear | Yes | Yes | Yes | Yes | No | Yes | Yes | Yes | Yes | Yes | Yes | Yes | N/A | Yes | No | Unclear | Yes | Low |
| **Russell 2023** | Unclear | Yes | Yes | Yes | Yes | No | Yes | Yes | Yes | Yes | Yes | Yes | Yes | N/A | Yes | Unclear | Unclear | Yes | Low |
| **Grando 2022** | Unclear | Yes | Yes | Unclear | Yes | No | No | Yes | Yes | Yes | Yes | Yes | Yes | N/A | Yes | No | Unclear | Yes | Moderate |
| **Miles-Cohen, 2020** | Unclear | Yes | Yes | Unclear | Yes | Yes | Unclear | Yes | Yes | Yes | Yes | Yes | Yes | N/A | Unclear | No | Unclear | Yes | Moderate |
| **Sears 2019** | Unclear | Yes | Yes | Yes | Yes | No | Unclear | Yes | Unclear | Yes | Yes | Yes | Yes | N/A | Yes | No | Unclear | Yes | Moderate |
| **Parker 2018** | Unclear | Unclear | Unclear | Unclear | Unclear | No | Unclear | Yes | Unclear | Yes | Yes | Yes | Yes | N/A | Yes | Yes | Unclear | Yes | High |
| **Schwartz 2021** | Unclear | Yes | Yes | Yes | Yes | Yes | Yes | Yes | Yes | Yes | Yes | Yes | Yes | N/A | Yes | No | Unclear | Yes | Low |
| **Deutsch 2022** | Unclear | Yes | Yes | Yes | Yes | Unclear | Unclear | Yes | Yes | Yes | Yes | Yes | Yes | N/A | Yes | No | Unclear | Yes | Low |

## Part 2: Studies appraised using the JBI qualitative and quasi-experimental checklists

| **Study** | **Q1. Philosophical congruity with methodology** | **Q2. Methodology-question congruity** | **Q3. Methodology-data collection congruity** | **Q4. Methodology-analysis congruity** | **Q5. Methodology-interpretation congruity** | **Q6. Researcher positionality stated** | **Q7. Researcher influence addressed** | **Q8. Participant voices represented** | **Q9. Ethical approval** | **Q10. Conclusions follow from data** | **QE1. Cause and effect clearly defined** | **QE2. Comparison groups similar** | **QE3. Similar co-interventions** | **QE4. Control group present** | **QE5. Multiple outcome measurements** | **QE6. Follow-up complete** | **QE7. Outcomes measured consistently** | **QE8. Outcomes measured reliably** | **QE9. Appropriate statistical analysis** | **Overall appraisal rating** |
| --- | --- | --- | --- | --- | --- | --- | --- | --- | --- | --- | --- | --- | --- | --- | --- | --- | --- | --- | --- | --- |
| **Hopkins 2022** | Unclear | Yes | Yes | Yes | Yes | No | No | Yes | Unclear | Yes | Yes | Yes | N/A | No | No | No | Yes | Unclear | Yes | Moderate |
| **Sims 2022** | Unclear | Yes | Yes | Yes | Yes | No | No | Yes | Unclear | Yes | Yes | Yes | N/A | No | No | No | Yes | Unclear | Yes | Moderate |
| **Barnard 2022** | Unclear | Yes | Yes | Yes | Yes | No | No | Yes | Unclear | Yes | Yes | No | Unclear | Yes | No | No | Yes | Unclear | Yes | Moderate |
| **Danhauer 2019** | Unclear | Yes | Unclear | No | Yes | No | No | Yes | Yes | Yes | Yes | Yes | N/A | No | No | Yes | Yes | Unclear | Yes | Moderate |
| **Lee 2024** | Unclear | Yes | Yes | Yes | Yes | Yes | Unclear | Yes | Yes | Yes | Yes | Yes | N/A | No | No | Yes | Yes | Unclear | Yes | Low |
| **DeFrank-Cole 2017** | Yes | Yes | Yes | Yes | Yes | Unclear | Unclear | Yes | Yes | Yes | Yes | Yes | N/A | No | No | Unclear | Yes | Unclear | Yes | Moderate |
| **Herbst 2024** | Yes | Yes | Yes | Yes | Yes | Unclear | Unclear | Yes | Yes | Yes | Yes | Yes | N/A | No | No | Yes | Yes | Yes | Yes | Low |
| **Drake 2023** | Unclear | Unclear | Unclear | Unclear | Unclear | No | No | Yes | Unclear | Unclear | Unclear | Yes | N/A | No | No | Unclear | Unclear | Unclear | Unclear | High |

***Note****.* Risk of bias was rated low (≥70% of applicable items scored ‘yes’), moderate (50-69%), or high (≤49%). N/A items were excluded from the denominator.

# Supplementary Table S8F. Quality appraisal: Descriptive reports / text and opinion (JBI Checklist)

| **Study** | **1. Source clearly identifiable** | **2. Source has relevant expertise** | **3. Patient/client interests central** | **4. Opinion logically consistent** | **5. Reasoning well-argued** | **6. Reference to existing literature** | **Overall appraisal rating** |
| --- | --- | --- | --- | --- | --- | --- | --- |
| **Mohanakumar 2022** | Yes | Yes | Yes | Yes | Yes | Yes | Low |
| **Van Oosten 2017** | Yes | Yes | Yes | Unclear | Yes | Yes | Low |

***Note****.* Risk of bias was rated low (≥70% of applicable items scored ‘yes’), moderate (50-69%), or high (≤49%). N/A items were excluded from the denominato

# Supplementary Table S9. Reference list of all included studies

1. Adeola MT, Fink AM, Shen Q, et al. Growing the next generation of nurse leaders: a report of the process and outcomes of the Midwest Nursing Research Society Leadership Academy. West J Nurs Res 2024; 46: 478–82.

2. Barnard S, Arnold J, Bosley S, Munir F. The personal and institutional impacts of a mass participation leadership programme for women working in higher education: a longitudinal analysis. Stud High Educ 2022; 47: 1372–85.

3. Brixey AG, Spalluto LB, DeWeese L. Launching a women-focused faculty and trainee development program. Acad Radiol 2025; 32: 4829–38.

4. Brue KL, Brue SA. Experiences and outcomes of a women’s leadership-development program: a phenomenological investigation. J Leadersh Educ 2016; 15: 75–97.

5. Brue KL, Brue SA. Leadership role identity construction in women’s leadership development programs. J Leadersh Educ 2018; 17: 7–27

6. Chang S, Morahan PS, Magrane D, et al. Retaining faculty in academic medicine: the impact of career development programs for women. J Womens Health 2016; 25: 687–96.

7. Chang S, Guindani M, Morahan P, Magrane D, Newbill S, Helitzer D. Increasing promotion of women faculty in academic medicine: impact of national career development programs. J Womens Health 2020; 29: 837–46.

8. Chasserio S, Bacha E. Women-only training programmes as tools for professional development: analysis and outcomes of a transformative learning process. Eur J Train Dev 2024; 48: 455–77.

9. Chaudron LH, Anson E, Bryson Tolbert JM, Inoue S, Cerulli C. Meeting the needs of mid-career women in academic medicine: one model career development program. J Womens Health 2021; 30: 45–51.

10. Cordeiro AA, Walsh KF, Sundararajan R, et al. The Female Global Scholars Program: a mixed-methods evaluation of a novel intervention to promote the retention and advancement of women in global health research. PLOS Glob Public Health 2024; 4: e0002974.

11. Danhauer SC, Tooze JA, Barrett NA, et al. Development of an innovative career development program for early-career women faculty. Glob Adv Health Med 2019; 8: 2164956119862986.

12. David DH, Arora IS, Hussein A, Santana JG, Crusto CA, Latimore D. Women’s leadership development at the Yale School of Medicine: preliminary evaluation of an innovative program. Womens Health Rep 2025; 6: 274–85.

13. DeFrank-Cole L, Latimer M, Neidermeyer P, Wheatly M. Understanding “why” one university’s women’s leadership development strategies are so effective. Adv Women Leadersh J 2017; 36: 26–35.

14. Deutsch N, Yanofsky SD, Markowitz SD, et al. Evaluating the Women’s Empowerment and Leadership Initiative: supporting mentorship, career satisfaction, and well-being among pediatric anesthesiologists. Paediatr Anaesth 2022; 33: 6–16.

15. Drake AF, Sollecito WA, Horneffer KE, et al. Building diverse leadership in an academic medical center: the ACCLAIM program. J Natl Med Assoc 2023; 115: 134–43.

16. Fassiotto M, Jerome B, Stefanac L, et al. Stanford Network for Advancement and Promotion: the impact of a community building-focused leadership development program on the success of underrepresented groups in academic medicine. Health Care Manage Rev 2024; 49: 272–80.

17. Ford AY, Dannels S, Morahan P, Magrane D. Leadership programs for academic women: building self-efficacy and organizational leadership capacity. J Womens Health 2021; 30: 672–80.

18. Grando A, Ancker JS, Tao D, et al. Design and evaluation of a Women in American Medical Informatics Association (AMIA) leadership program. J Am Med Inform Assoc 2022; 29: 164–70.

19. Heilemann B, Parker P. Building career capital in policing in Australia through women’s leadership programmes. Polic Int J 2024; 47: 95–111.

20. Helitzer DL, Newbill SL, Cardinali G, et al. Narratives of participants in national career development programs for women in academic medicine: identifying the opportunities for strategic investment. J Womens Health 2016; 25: 360–70.

21. Herbst THH, Roux AT, Naidoo V. The year I found my voice: transforming self-confidence through a women’s leadership programme. Transform High Educ 2024; 9: a438.

22. Hopkins K, Meyer M, Afkinich J, Bialobrzeski E, Perry V. Impact of leadership development and facilitated peer coaching on women’s individual, collective, and organizational behaviors in human services. Nonprofit Manag Leadersh 2022; 32: 387–408.

23. Jones RD, Lin YJ, Thomas JD, et al. Participant experiences with EMPOWER: benefits, barriers, and best practices for an online peer mentorship and leadership program for women in academic medicine. Womens Health Rep 2025; 6: 26884844251403443.

24. Jowett S, Slade K, Gosai J, Davis L. Women coaches leadership development programme: an evaluation study of programme effectiveness. Front Sports Act Living 2024; 6: 1433787.

25. Kang JY, Croghan IT, Matchett CL, et al. Reflect, Inspire, Strengthen, and Empower 2.0 Program: advancing careers and leadership for women physician staff in an academic institution. Womens Health Rep 2024; 5: 65–74.

26. Kelly EH, Miskimen T, Rivera F, Peterson LE, Hingle ST. Women’s Wellness Through Equity and Leadership (WEL): a program evaluation. Pediatrics 2021; 148 (suppl 2): e2021051440I.

27. Knipfer K, Shaughnessy B, Hentschel T, Schmid E. Unlocking women’s leadership potential: a curricular example for developing female leaders in academia. J Manag Educ 2017; 41: 272–302.

28. Kvach E, Yesehak B, Abebaw H, Conniff J, Busse H, Haq C. Perspectives of female medical faculty in Ethiopia on a leadership fellowship program. Int J Med Educ 2017; 8: 314–23.

29. Lämsä AM, Savela T. The effects of leadership development on women’s career success. Int J Hum Resour Dev Manag 2019; 19: 21–36.

30. Lee J, Tackett S, Skarupski K, et al. Inspiring and preparing our future leaders: evaluating the impact of the Early Career Women’s Leadership Program. J Healthc Leadersh 2024; 16: 287–302.

31. Martínez-Martínez M, Molina-López MM, de Cabo RM, et al. Awakenings: an authentic leadership development program to break the glass ceiling. Sustainability 2021; 13: 7476.

32. Mbilishaka A, Vixamar-Owens DZ, Fredericks A, Massey A. Dialogues in Leadership Herstory: exploring the experiences of Black women faculty in a leadership-development program. Consult Psychol J 2023; 75: 119–34.

33. Megheirkouni M, Roomi MA. Women’s leadership development in sport settings: factors influencing the transformational learning experience of female managers. Eur J Train Dev 2017; 41: 467–84.

34. Miles-Cohen SE, Brown KS, Shullman SL, Coons HL. American Psychological Association Leadership Institute for Women in Psychology: herstory, impacts, and next steps. Psychol Manag J 2020; 23: 139–62.

35. Mohanakumar S, Dananjanie D, Samsudin SSB, Lan HTM, Sabree S. Reflections on the Container Control Programme–Women’s Professional Development Programme: transforming women’s leadership in customs administrations. World Customs J 2022; 16: 157–64.

36. Nakamura YT, Hinshaw J, Burns R. Developing women’s authenticity in leadership. Merits 2022; 2: 408–26.

37. Nash M, Moore R. An evaluation of a leadership development programme for women in STEMM in Antarctica. Polar J 2018; 8: 110–25.

38. Nash M, Moore R. ‘When I look in the bathroom mirror, I see all the women standing behind me’: an evaluation of a leadership development program for women in STEMM. J Sociol 2021; 57: 362–79.

39. Nowling TK, McClure E, Simpson A, Sheidow AJ, Shaw D, Feghali-Bostwick C. A focused career development program for women faculty at an academic medical center. J Womens Health 2018; 27: 1474–81.

40. O’Brien K, Allin L. Transformational learning through a women’s outdoor leadership course. J Adventure Educ Outdoor Learn 2022; 22: 191–202.

41. Parker P, Hewitt B, Witheriff J, Cooper A. Frank and fearless: supporting academic career progression for women in an Australian program. Adm Sci 2018; 8: 5.

42. Pelfrey CM, Cola PA, Gerlick JA, Edgar BK, Khatri SB. Breaking through barriers: factors that influence behavior change toward leadership for women in academic medicine. Front Psychol 2022; 13: 854488.

43. Peterson H. A women-only leadership development program: facilitating access to authority for women in Swedish higher education? Soc Sci 2019; 8: 137.

44. Round H, Yamao S, Shao B, Klonek F, Sekiguchi T, Newman A. Fostering psychological capital self-efficacy in emerging female leaders: practical insights from an international leadership development program. Organ Dyn 2024; 53: 101085.

45. Russell M, Stewart B, Brooks L. Advancing gender equity and women’s leadership capacity: mentoring, networking, and training. Adv Women Leadersh J 2023; 42: 88–97.

46. Ryan NF, Hammond M, MacCurtain S, Cross C. Overcoming risk for women in leader identity development. J Manag Psychol 2021; 36: 75–88.

47. Schwartz JM, Markowitz SD, Yanofsky SD, et al. Empowering women as leaders in pediatric anesthesiology: methodology, lessons, and early outcomes of a national initiative. Anesth Analg 2021; 133: 1497–509.

48. Sears D, White BAA, Dewsnap M, Janek H. Development and implementation of the Women Leaders in Medicine Program at a multispecialty health care system. Proc Bayl Univ Med Cent 2019; 32: 477–80.

49. Sears DM, Bejcek A, Kilpatrick L, et al. Leadership development as a novel strategy to mitigate burnout among female physicians. PLoS One 2025; 20: e0319895.

50. Selzer R, Howton A, Wallace F. Rethinking women’s leadership development: voices from the trenches. Adm Sci 2017; 7: 18.

51. Sims CM, Carter AD, Frady KK, et al. Furthering women faculty in leadership roles: a human performance improvement case study. Perform Improv Q 2022; 34: 395–426.

52. Skarupski KA, Levine RB, Rand C, González-Fernández M, Clements JE, Fivush B. Impact of a leadership program for women faculty: a retrospective survey of eight years of cohort participants. J Fac Dev 2019; 33: 7–14

53. Spalluto LB, Spottswood SE, Deitte LA, Chern A, Dewey CM. A leadership intervention to further the training of female faculty (LIFT-OFF) in radiology. Acad Radiol 2017; 24: 709–16.

54. Sutton S, Palmisano A, Ginsburg E. A coaching-based leadership program for women postdoctoral fellows at the National Cancer Institute that cultivates self-confidence and persistence in STEMM. J Cancer Educ 2024; 39: 507–19.

55. Valerio E, Spence R, Adapa S. Women’s leadership development in agricultural research: an empirical study in Southeast Asia and the Pacific. Cogent Educ 2024; 11: 2351240.

56. Van Oosten EB, Buse K, Bilimoria D. The Leadership Lab for Women: advancing and retaining women in STEM through professional development. Front Psychol 2017; 8: 2138.

57. Walsh KF, Fahme S, Reif LK, Mathad JS, Downs JA. Novel, low-cost intervention to promote women’s advancement in global health research. Acad Med 2022; 97: 84–89.

# Supplementary Table S10. Studies excluded at full-text screening and not retrieved, with reasons

| **#** | **Study** | **Reason for exclusion at full text** |
| --- | --- | --- |
| 1 | Desormeaux, 2023 | Focused on the coaching component within a women's career-advancement and leadership-development programme, rather than evaluating the leadership development programme as a whole. |
| 2 | Swan Dagen et al., 2022 | Described the Women's Leadership Initiative framework and examples of leadership-education opportunities, rather than reporting an empirical evaluation of programme outcomes. |
| 3 | Madden & Levenshus, 2021 | Evaluated a women's political leadership development programme, rather than a programme delivered to adult professionals in a formal organisational or workplace setting. |
| 4 | Mann, 2021 | Dissertation; not an eligible peer-reviewed journal article. |
| 5 | Sims et al., 2021 (Do servant, transformational, transactional, and passive avoidant leadership styles influence mentoring competencies for faculty?) | Examined associations between faculty members' leadership styles and mentoring competencies, rather than evaluating the outcomes of a leadership development programme. |
| 6 | Rowlands et al., 2020 | Examined graduates' experiences of leadership development programmes and impediments to organisational or industry change, rather than evaluating a clearly bounded programme with extractable participant-level outcomes. |
| 7 | Santillo, 2017 | Dissertation; not an eligible peer-reviewed journal article. |
| 8 | Levine et al., 2015 | Published before the review's September 2015 start date. |
| 9 | Alreshidi et al., 2024 | Evaluated a nursing leadership development programme, but eligible outcomes were not reported separately for women. |
| 10 | Zaleski, 2021 | Commentary on the Women's Empowerment and Leadership Initiative, rather than an empirical evaluation of programme outcomes. |
| 11 | Margolis et al., 2021 | Evaluated the GAIN networking pilot within WELI, rather than the leadership development initiative as a whole. |
| 12 | Lewis et al., 2021 | Evaluated institutional action projects associated with ELAM, rather than evaluating the ELAM leadership development programme as a whole. |
| 13 | Banister et al., 2020 | Evaluated a leadership development programme for African American RN graduates, but eligible outcomes were not reported separately for women. |
| 14 | Ramseur et al., 2018 | Evaluated a structured nursing leadership development programme, but eligible outcomes were not reported separately for women. |
| 15 | Watterston & Ehrich, 2024 | Reviewed literature and reflected on women's leadership development programmes run by the authors, rather than reporting an empirical evaluation of a specific programme. |
| 16 | Bond et al., 2023 | Evaluated leadership development programmes for nurses and midwives, but eligible outcomes were not reported separately for women. |
| 17 | Bond et al., 2022 | Evaluated a leadership development programme in the clinical learning environment, but eligible outcomes were not reported separately for women. |
| 18 | Lewis, 2023 | Explored gendered and racialised experiences within a leadership development programme, rather than reporting eligible programme outcome data for women. |
| 19 | Cox et al., 2022 | Book chapter; not an eligible peer-reviewed journal article. |
| 20 | Huang et al., 2022 | Evaluated nursing leadership training programmes, but eligible outcomes were not reported separately for women. |
| 21 | Ohlson et al., 2022 | Programme was delivered to university student-athletes, not adult professionals in formal organisational roles. |
| 22 | Sisk et al., 2021 | Evaluated an advanced leadership initiative for senior nurse leaders, but eligible outcomes were not reported separately for women. |
| 23 | Sims et al., 2021 (Advancing gender equity through mentoring and leadership development: A human performance technology case study) | Evaluated a mentoring and leadership development programme including mixed-gender faculty, but eligible outcomes were not reported separately for women. |
| 24 | Alfaraidy, 2021 | Source was not an eligible peer-reviewed journal article. |
| 25 | Häyrynen & Lämsä, 2021 | Evaluated leadership development for Finnish Defence Forces volunteers, not adult professionals in formal organisational roles. |
| 26 | Ahn & Cunningham, 2020 | Examined FIFA gender initiatives and glass-cliff dynamics; the female leadership development programme was part of the case context rather than the primary intervention evaluation. |
| 27 | Khalid et al., 2021 | Programme was evaluated among enrolled female students, not adult professionals in formal organisational roles. |
| 28 | Martins et al., 2019 | Developed and validated a leadership training programme, but did not evaluate programme effectiveness. |
| 29 | Ross-Davie et al., 2016 | Evaluated a midwifery leadership programme, but eligible outcomes were not reported separately for women. |
| 30 | Leitch et al., 2017 | Book chapter; not an eligible peer-reviewed journal article. |
| 31 | Solomon & Secrest, 2017 | Book chapter; not an eligible peer-reviewed journal article. |
| 32 | Longman et al., 2018 | Book chapter; not an eligible peer-reviewed journal article. |
| 33 | Pike et al., 2017 | Book chapter; not an eligible peer-reviewed journal article. |
| 34 | Shields & Cassada, 2016 | Examined access, equity, demographic profiles, and placement within a non-traditional leadership development programme, rather than evaluating participant leadership development outcomes. |
| 35 | Jones et al., 2023 | Evaluated a general public health leadership development programme, but eligible outcomes were not reported separately for women. |
| 36 | Greene et al., 2022 | Conference abstract only; not a full peer-reviewed journal article. |
| 37 | McGourty et al., 2020 | Conference abstract only; not a full peer-reviewed journal article. |
| 38 | Dirik & Intepeler, 2024 | Evaluated authentic leadership training for nurses/head nurses, but eligible outcomes were not reported separately for women. |
| 39 | Medero et al., 2023 | Evaluated a charge nurse leadership development programme, but eligible outcomes were not reported separately for women. |
| 40 | Tomanan et al., 2022 | Evaluated leadership training for public health nurses, but eligible outcomes were not reported separately for women. |
| 41 | Patterson et al., 2020 | Evaluated a nurse faculty leadership academy, but eligible outcomes were not reported separately for women. |
| 42 | Fitzpatrick et al., 2016 | Evaluated a clinical nurse leadership education and development programme, but eligible outcomes were not reported separately for women. |
| 43 | Culver et al., 2019 | Evaluated a social learning intervention for gender equity and leadership development, but did not report eligible participant-level leadership development outcomes for women. |
| 44 | Cutter et al., 2025 | Full text embargoed until 24 November 2026, beyond the review's search window. |
| 45 | Barbosa & Real de Oliveira, 2026 | Examined women entrepreneurs' leadership development journeys, rather than evaluating a defined leadership development programme. |
| 46 | Nahata et al., 2025 | Evaluated an intramural faculty leadership training programme, but eligible outcomes were not reported separately for women. |
| 47 | Cifre et al., 2025 | Evaluated a leadership intervention for young women in higher education before full labour-market entry, rather than adult professionals in formal organisational roles. |
| 48 | Williams et al., 2025 | Presented a framework or organisational development approach for advancing women leaders, rather than an empirical evaluation of a leadership development programme. |
| 49 | Mishkin et al., 2026 | Evaluated a brief preconference leadership workshop, rather than a structured leadership development programme. |
| 50 | Steele et al., 2024 | Examined women's professional organisations as contexts for leader development, rather than evaluating a defined leadership development programme. |
| 51 | Wheatly et al., 2023 | Reported an online pivot and increased offerings or participation in leadership education, rather than an eligible evaluation of participant leadership outcomes. |
| 52 | Carter et al., 2020 | Evaluated mentoring within a gender-equity leadership development programme, rather than the leadership development programme as a whole. |
| 53 | Finkel et al., 2023 | Analysed leadership training as a career-advancement factor in survey data, rather than evaluating a specific structured leadership development programme. |
| 54 | Kraft & Culver, 2021 | Evaluated action learning for four social learning leaders/facilitators within a broader women-in-sport initiative, rather than outcomes for participants in the leadership development programme. |
| 55 | Bilimoria & Singer, 2019 | Described and evaluated the IDEAL institutional gender-equity transformation initiative across universities, rather than evaluating a structured participant leadership development programme. |
| 56 | Box & Ellis, 2018 | Evaluated an action-learning intervention for women in higher education, rather than a clearly bounded leadership development programme with eligible outcome evaluation. |
| 57 | Yen et al., 2019 | Chronicled the growth and expansion of the ADVANCE leadership programme and toolkit, rather than reporting eligible participant-level outcome evaluation. |
| 58 | Yeh et al., 2025 | Evaluated empowering leadership training in long-term care settings, but eligible outcomes were not reported separately for women. |
| 59 | LaForest & Jadunandan, 2023 | Described the launch and purpose of the Black Nurses Leadership Institute, without reporting eligible programme outcome evaluation. |
| 60 | Himstedt et al., 2023 | Programme description without systematic evaluative outcome data. |
| 61 | Mucheru et al., 2024 | Review article; not a primary evaluation of a leadership or career advancement intervention. |
| 62 | Timbo et al., 2025 | Described creation of the Women Leaders in Eye Health initiative, rather than reporting an outcome evaluation of an implemented leadership development programme. |
| 63 | Iyer et al., 2025 | Described development of a collaborative for women in medicine and biomedical science, rather than reporting evaluative outcomes from a leadership development programme. |
| 64 | Magnus et al., 2019 | Book/report chapter; not an eligible peer-reviewed journal article. |
| 65 | Kalbarczyk et al., 2022 | Open-attendance virtual speaker series and near-peer networking platform, designed for and attended primarily by doctoral and masters students (59% of attendees). |
| 66 | Seale et al., 2021 | Reported a needs analysis and programme design response, rather than outcomes from an implemented leadership development programme. |
| 67 | Gomes et al., 2023 | Source was not an eligible peer-reviewed journal article. |
| 68 | Obi et al., 2026 | Examined perceptions of intersectional data practices and perceived leadership interventions, rather than evaluating an implemented leadership development programme. |
| 69 | Jones & Abdo, 2025 | Analysed Islamic leadership programmes for women conceptually, rather than reporting empirical outcome evaluation of a defined programme. |
| 70 | Debebe, 2017 | Examined women's leadership development transformations, rather than reporting eligible outcome data from a clearly defined programme evaluation. |
| 71 | Debebe et al., 2016 | Introduction/editorial to a special issue, not an empirical programme evaluation. |
| 72 | Tung et al., 2021 | Evaluated a general faculty development/leadership programme, but eligible outcomes were not reported separately for women. |
| 73 | Helitzer et al., 2017 | Focused on cultural change and critical actors associated with programme participation, rather than evaluating the leadership development programme itself. |
| 74 | Gronowski & Burnham, 2018 | Described a women's professional development programme rather than a structured leadership development programme. |
| 75 | Li-Sauerwine et al., 2022 | Evaluated a women's professional development programme for faculty and residents in emergency medicine, rather than a leadership development programme as the primary intervention. |
| 76 | Dyson et al., 2025 | Evaluated a women's research excellence/career development intervention rather than a leadership development programme with eligible leadership outcomes. |
| 77 | Fassinger et al., 2025 | Traced the history and feminist leadership principles of the Leadership Institute for Women in Psychology, rather than reporting an empirical programme outcome evaluation. |
| 78 | Kassotakis & Rizk, 2015 | Book chapter; not an eligible peer-reviewed journal article. |
| 79 | Pololi et al., 2025 | Evaluated a dialogue exercise embedded in a mentoring and leadership programme, rather than evaluating the programme as a whole. |
| 80 | Clark et al., 2023 | Evaluated an online leadership curriculum for midwives, but eligible outcomes were not reported separately for women. |
| 81 | Chuang, 2019 | Explored women-only training for professional development, rather than reporting an eligible evaluation of a leadership development programme. |
| 82 | Böhmer & Schinnenburg, 2018 | Focused on future female leaders before labour-market entry and proposed teaching/workshop approaches, rather than evaluating a structured leadership development programme for adult professionals. |
| 83 | Kelinsky & Anderson, 2016 | Leadership training was delivered to volunteer mentors in a youth camp context, rather than to adult professionals in a formal organisational setting. |
| 84 | Gaetke-Udager et al., 2018 | Evaluated a Women in Radiology group rather than a structured leadership development programme. |
| 85 | Girod et al., 2016 | Evaluated a brief implicit-bias education session, not a structured leadership development programme. |
| 86 | Gregory-Smith, 2018 | Evaluated Athena SWAN as an institutional gender-equity charter/positive-action policy, rather than a structured leadership development programme. |
| 87 | Grisso et al., 2017 | Evaluated a multifaceted career-success intervention for women assistant professors, rather than a structured leadership development programme as the primary intervention. |
| 88 | Tomblinson et al., 2022 | Evaluated a Women in Radiology programme focused on workplace gender equity, career satisfaction, mentorship, work-life balance, teaching, and research, rather than leadership development as the primary programme aim. |
| 89 | Fitzpatrick et al., 2025 | Examined the experiences and career trajectories of underrepresented physician trainees within addiction medicine training programmes, rather than evaluating a structured leadership development programme. |
| 90 | An & Meier, 2021 | Evaluated generic (mixed-sex) transformational and transactional leadership training with no women-specific or gender-equity component; sex was examined as a moderator of a gender-neutral intervention, which falls outside the review's intervention criterion. |
| 91 | Ribera et al., 2026 | Evaluated a generic early-career faculty leadership programme open to all faculty, with no women-specific or gender-equity component; outcomes for women were derived from sex-disaggregated subgroup analyses within a mixed-sex propensity-matched sample, outside the review's intervention criterion. |
| 92 | Fassiotto et al., 2018 | Evaluated a generic physician leadership development programme with no women-specific or gender-equity component; women's outcomes arose from demographic subgroup interactions within a mixed-sex sample, outside the review's intervention criterion. |
